# *Brucella melitensis *global gene expression study provides novel information on growth phase-specific gene regulation with potential insights for understanding *Brucella*:host initial interactions

**DOI:** 10.1186/1471-2180-9-81

**Published:** 2009-05-06

**Authors:** Carlos A Rossetti, Cristi L Galindo, Sara D Lawhon, Harold R Garner, L Garry Adams

**Affiliations:** 1 Department of Veterinary Pathobiology, College of Veterinary Medicine & Biomedical Sciences, Texas A&M University, College Station, TX 77483-4467, USA; 2 Departments of Biochemistry and Internal Medicine, University of Texas Southwestern Medical School, Dallas, TX 75390, USA; 3 Instituto de Patobiología, CICVyA-CNIA, INTA. CC25, (B1712WAA), Castelar, Buenos Aires, Argentina

## Abstract

**Background:**

*Brucella *spp. are the etiological agents of brucellosis, a zoonotic infectious disease that causes abortion in animals and chronic debilitating illness in humans. Natural *Brucella *infections occur primarily through an incompletely defined mechanism of adhesion to and penetration of mucosal epithelium. In this study, we characterized changes in genome-wide transcript abundance of the most and the least invasive growth phases of *B. melitensis *cultures to HeLa cells, as a preliminary approach for identifying candidate pathogen genes involved in invasion of epithelial cells.

**Results:**

*B. melitensis *at the late logarithmic phase of growth are more invasive to HeLa cells than mid-logarithmic or stationary growth phases. Microarray analysis of *B. melitensis *gene expression identified 414 up- and 40 down-regulated genes in late-log growth phase (the most invasive culture) compared to the stationary growth phase (the least invasive culture). As expected, the majority of up-regulated genes in late-log phase cultures were those associated with growth, including DNA replication, transcription, translation, intermediate metabolism, energy production and conversion, membrane transport, and biogenesis of the cell envelope and outer membrane; while the down-regulated genes were distributed among several functional categories.

**Conclusion:**

This *Brucella *global expression profile study provides novel information on growth phase-specific gene expression. Further characterization of some genes found differentially expressed in the most invasive culture will likely bring new insights into the initial molecular interactions between *Brucella *and its host.

## Background

Bacteria from the genus *Brucella *are the etiological agents of brucellosis, a worldwide zoonotic infectious disease that has a negative economic impact on animal production and human public health [1,2]. Based on its 16S rRNA sequence, *Brucella *is included in the α2 subclass of the Proteobacteria, along with plant (*Agrobacterium *and the Rhizobiaceae) and other mammalian (*Bartonella *and the Rickettsiae) symbionts [3]. The genus *Brucella *consists of six recognized species, grouped according to their primary host preferences, i.e. *B. abortus *: cattle, *B. melitensis *: sheep and goats, *B. suis *: hogs, *B. ovis *: sheep, *B. canis *: dogs and *B. neotomae *: wood desert rats [4]. Due to their high virulence to humans, *B. abortus, B. melitensis *and *B. suis *are considered potential bioterrorist agents, having been classified as major biodefense/biothreat pathogens, and their possession and use is strictly regulated in the United States [5].

Natural *Brucella *infections occur primarily through adhesion to and penetration of mucosal epithelia. The mucosal surface of the alimentary tract is a major route for *B. melitensis *and *B. abortus *invasion, while the mucosa of the genital tract is the principal route of entry for *B. ovis*, *B. suis *and *B. canis *[4,6]. *In vitro *studies have shown that within a few minutes after binding non-professional phagocytic cells, *Brucella *are actively internalized via receptor-mediated phagocytosis without inducing obvious damage to the cells [7,8]. *Brucella *bind sialic acid residues present on eukaryotic cell membranes [9] and are internalized by epitheloid-like cells in an active mechanism in which the organism induces its own internalization via activation of small GTPases of the Rho subfamily and rearrangements of the host cell actin cytoskeleton and microtubules [10].

Bacteria have the ability to express surface molecules able to recognize unique or common receptor components present on many eukaryotic cell surface. Three *Brucella *gene products have been characterized as important for invasion in non-phagocytic cells: a two-component regulatory system (BvrR/BvrS) that modulates the expression of outer membrane proteins necessary for recruiting small GTPase proteins required for actin polymerization and penetration [11,12], a *Brucella *surface protein, called SP41, which enables *Brucella *to adhere to non-phagocytic cells [13], and a hypothetical protein encoded by the BMEI0216 gene, which is critical for *Brucella melitensis *internalization in HeLa cells after 1 h post-infection [14]. These few examples are all that is currently known about the molecular mechanisms underlying *Brucella *adhesion and internalization in eukaryotic cells. HeLa cells have extensively been used as a model to investigate the internalization of brucellae of epithelial cells during the colonization of the susceptible host [9,10]. Here, we employed this cell line to evaluate the rate of invasion of *B. melitensis *at different growth phases. Our results indicate that cultures of *B. melitensis *in the late-log phase of growth were more invasive in non-professional phagocytic cells than cultures at mid-log and stationary growth phases. Using cDNA microarrays, we characterized the transcriptome of the most (late-log) and the least (stationary) invasive growth phases of *B. melitensis *cultures as a preliminary approach for identifying pathogen candidate genes involved in epithelial cell invasion process. Microarray analysis revealed a greater number of genes up-regulated in these cultures than in stationary phase cultures. Consistent with the expected differences due to growth, there was a more active metabolism and invasiveness of cultures in late-log phase than cultures in stationary phase. Given the role that some of these genes have in pathogenesis in other bacterial species, we believe that these data may offer insight into potential growth-phase regulated *Brucella *virulence genes involved in the initial host:pathogen interactions.

## Results

### *B. melitensis *16 M at late-log phase of growth were more invasive to epithelial cells than were bacteria at mid-log and stationary growth phases

**Figure 1:**
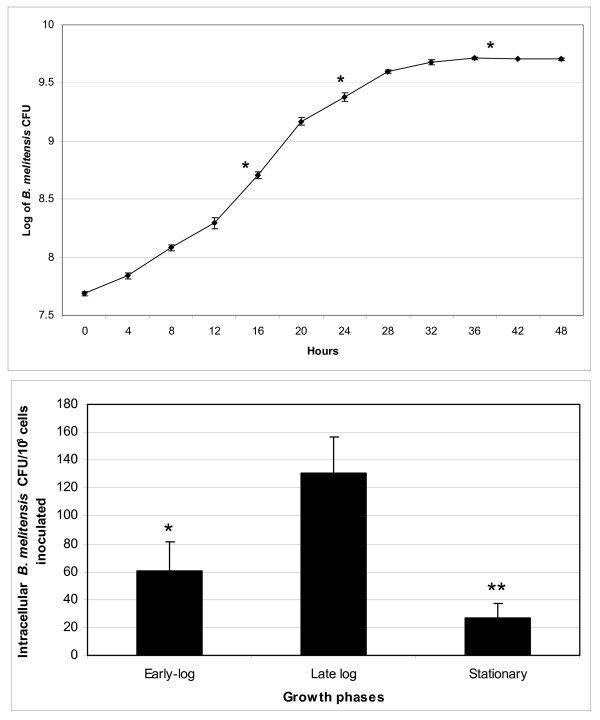
**The ability of *B. melitensis *16 M at different phases of growth to invade HeLa cells**. **(A) **Growth curve of *B. melitensis *16 M grown overnight in tubes with loose lids and shaking in F12K cell culture medium supplemented with 10% (v/v) HI-FBS. Results are the average +/- SD of 3 independent experiments. Mid-log, late-log and stationary growth phases are marked with *. **(B) **HeLa cell infections were performed at MOI 1,000:1 for 30 min. The intracellular number of late-log growth phase cultures of *B. melitensis *was significantly different from those grown to mid-log (* = *P *< 0.05) and stationary (** = *P *< 0.01) growth phases. Results are presented as the number of CFU from internalized bacteria 30 min post-infection per 10^3 ^cells inoculated. Data presented are the mean +/- SD (error bars) of triplicate samples from 3 independent experiments.

As described in the Methods section, *B. melitensis *was grown to mid-log growth phase, late-log growth phase, or stationary growth phase. At each of these growth phases, bacteria were enumerated, used to infect a representative epithelial cell line (HeLa cells), and RNA was extracted and microarrays were performed to identify altered gene expression. Under our experimental conditions, there were 0.5 × 10^9 ^CFU/ml (OD = 0.18) at the mid-log growth phase, 2 × 10^9 ^CFU/ml (OD = 0.4) at late-log phase, and 5 × 10^9 ^CFU/ml (OD = 0.72) at stationary phase (Figure 1A). For invasion experiments, a consistent multiplicity of infection (MOI) factor of 1,000 *B. melitensis *cells per HeLa cell was used to normalize the number of bacteria used. The average number of intracellular bacteria recovered was 60 CFU at mid-log phase, 130 CFU at late-log phase of growth and 27 CFU at stationary growth phase per 10^3 ^cells inoculated (Figure 1B). These values represent the average of three independent experiments. *B. melitensis *16 M cultures grown to late-log phase and then co-incubated with HeLa cells for 30 min were therefore 2.2 (*P *< 0.05) and 4.8 (*P *< 0.01) times more invasive than were cultures at mid-log and stationary growth phases.

### Whole-genome expression analysis of the most and the least *B. melitensis *16 M invasive growth phases: Reliability of array data

To analyze the molecular differences between the most and the least invasive phenotype, four biological replicates of cultures at late-log and stationary growth phases were analyzed using cDNA microarrays. Genomic DNA was used as an internal control for each experiment in order to allow experiment-to-experiment comparisons [15]. As expected, there was little variability between gDNA signals from array to array, even under the two different conditions examined (i.e., late-log and stationary growth phases). The R^2 ^value for any two arrays (for gDNA Cy5 fluorescent values) was between 0.78 and 0.89, even before normalization. When the values for each conditional replicate were averaged (four arrays each for late-log phase and stationary growth phases), the resulting R^2 ^value was 0.88 [see Additional file 1]. Comparisons of RNA Cy3 fluorescent signals (late-log versus late-log phases and stationary versus stationary phases) yielded similar R^2 ^values (data not shown).

**Figure 2:**
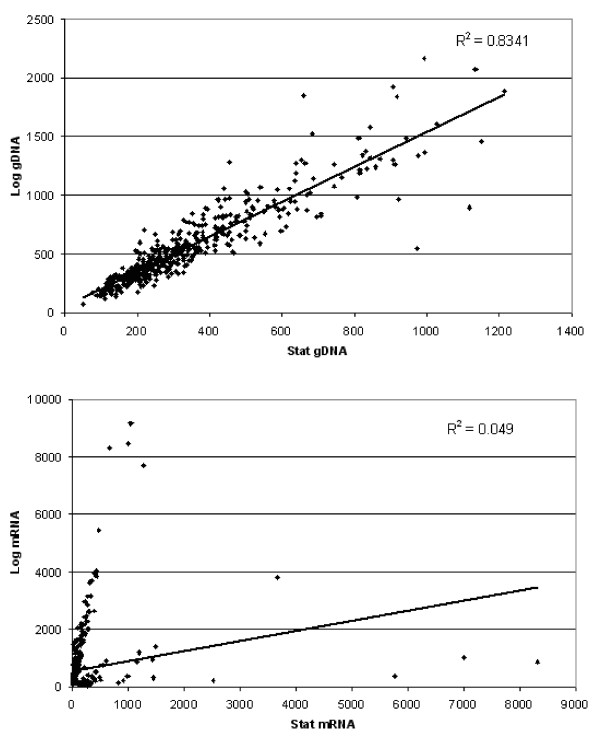
**Fluorescent signal values of *B. melitensis *transcript or gDNA from differentially expressed genes at stationary and late-log phases of growth**. Average Cy5 (gDNA) or Cy3 (transcript) signal values for *B. melitensis *grown in F12K tissue culture medium to late-log and stationary phases (4 arrays each) were plotted in Excel. Each dot represents the signal value for an individual spot on the array, determined to be significantly differentially expressed between late-log and stationary phases. **(A) **Comparison of genomic DNA levels of significant genes at stationary and late-log phases of growth. Stationary phase gDNA signal values are on the ordinate, and late-log phase signal values are on the abscissa. The R-squared value (0.8341) is displayed in the upper right-hand quadrant of the graph. **(B)**. Comparison of transcript levels of significant genes at stationary and late-log phases of growth. Stationary phase transcript signal values are on the ordinate, and late-log phase signal values are on the abscissa. Note the very low R-squared value (0.049), displayed in the upper right-hand quadrant of the graph. Stat refers to stationary phase, log refers to mid-log phase, and gDNA refers to genomic DNA.

In order to further minimize the incidence of false positives and increase the consistency and reliability of the microarray analysis results, the data were analyzed separately using four different techniques: GeneSpring combinatorial analysis, Spotfire DecisionSite 8.2 pairwise comparisons, SAM two-class unpaired comparisons, and ANOVA. A change in gene expression was considered significant if the *P *value was less than 0.05, the fold-change was at least 2.0, and the gene expression alteration occurred for all replicate experiments. We further expected each gene to be significantly differentially expressed for at least two of the three replicate spots for each experimental array set (stationary versus late-log phases). Based on these criteria, genes that were deemed significant by all four analytical methods (GeneSpring, Spotfire DecisionSite 8.2, SAM, and ANOVA) were organized by COGs functional categories [16] and compiled into a list that included 454 genes (different loci) that were up- or down-regulated when *B*. *melitensis *was grown to late-log phase, compared to stationary phase [see Additional file 2]. A direct comparison of the signal intensity values of these genes indicated that the difference between log and stationary phases was specifically due to differential gene expression and not array spatial bias, as indicated in Figure 2. When the average gDNA intensity values for these 454 genes were plotted (stationary phase versus late-log phase), the R^2 ^value was 0.83 (Figure 2A). However, the R^2 ^value for the same genes comparing the Cy3 fluorescence values instead (labeled cDNA amplified from RNA) was extremely low (R^2 ^= 0.049, Figure 2B).

**Figure 3:**
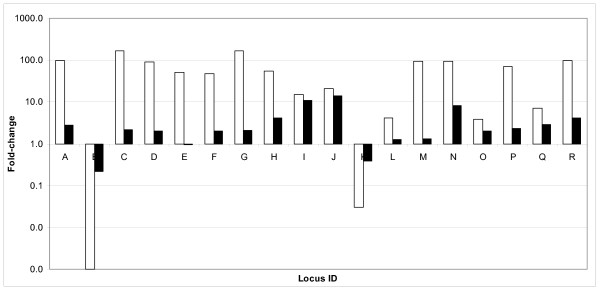
**Validation of DNA microarray results by quantitative RT-PCR**. Eighteen randomly selected ORFs that were differentially expressed based on microarray analysis between late-log and stationary growth phase were validated by quantitative RT-PCR. Seventeen of 18 ORFs tested showed fold-changes in the same direction by both methodologies and 15 of them were also altered greater than 2-fold. Functional classifications are as follows (tested loci in parentheses): A, DNA replication, recombination and repair (BMEII0663); B, Transcription (BMEI1384); C, Translation, ribosomal structure and biogenesis (BMEI1798); D, Nucleotide metabolism (BMEI0608); E, Carbohydrate metabolism (BMEI0344); F, Lipid metabolism (BMEII0047); G, Amino acid metabolism (BMEI0730); H, Secondary metabolites biosynthesis, transport and metabolism (BMEII0079); I, Energy production and conversion (BMEI0475); J, Inorganic ion transport and metabolism (BMEII1120); K, Cofactor transport and metabolism (BMEI0842); L, Cell envelope, biogenesis and outer membrane (BMEI0402); M, Membrane transport (BMEI0642); N, Defense mechanism (BMEII0382); O, Signal transduction (BMEI2034); P, Post-translational modification and secretion, protein turnover and chaperones (BMEI0645); Q, Cell division (BMEI0073); R, Cell motility and chemotaxis (BMEII0150). Open bars indicate microarray fold-change, solid bars indicate qRT-PCR fold-change.

To confirm the microarray results, we randomly chose 18 differentially expressed genes (one from each COGs functional category) and performed qRT-PCR. Based on qRT-PCR results, transcript levels of 15 of these genes (83%) were altered greater than 2.0-fold and in the same direction as was determined by microarray analysis. Two other genes (BMEI0402 and BMEI0642) were determined to be differentially expressed and in the same direction of microarray analysis, but the fold change was lower than 2. No significant difference in the expression level of BMEI0344 was observed by qRT-PCR (Figure 3).

### *B. melitensis *16 M express different sets of genes in late-log and stationary phases of growth in F12K tissue culture medium

**Figure 4:**
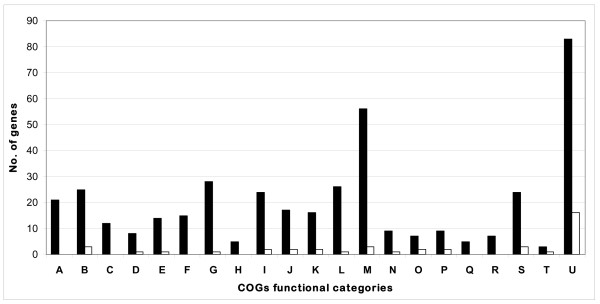
**Distribution of genes differentially expressed at late-log growth phase compared to stationary phase associated in cluster of ortholog genes (COGs) functional categories**. Functional classifications are as follows: A, DNA replication, recombination and repair; B, Transcription; C, Translation, ribosomal structure and biogenesis; D, Nucleotide metabolism; E, Carbohydrate metabolism; F, Lipid metabolism; G, Amino acid metabolism; H, Secondary metabolites biosynthesis, transport and metabolism; I, Energy production and conversion; J, Inorganic ion transport and metabolism; K, Cofactor transport and metabolism; L, Cell envelope, biogenesis and outer membrane; M, Membrane transport; N, Defense mechanism; O, Signal transduction; P, Post-translational modification and secretion, protein turnover and chaperones; Q, Cell division; R, Cell motility and chemotaxis; S, General function prediction only; T, Predicted by homology; U, Unknown function. Solid bars, up-regulated genes; open bars, down-regulated genes.

Of the 454 genes significantly altered in *B*. *melitensis *during late-log phase (14% of *B. melitensis *genome), 414 (91%) were up- and 40 (9%) were down-regulated, compared to when the bacteria were allowed to reach stationary phase [see Additional file 2]. The relative changes in gene expression ranged from a 386.5-fold induction of the Glycerol-3-phosphate regulon repressor gene (BMEII1093) to a 60.5-fold down-regulation of the locus BMEII0615 (hypothetical protein). As expected, the majority of gene expression changes were associated with growth and metabolism. Among the up-regulated genes were those associated with DNA replication, transcription and translation (57 genes), nucleotide, amino acid, lipid and carbohydrate metabolism (65 genes), energy production and conversion (24 genes), membrane transport (56 genes) and cell envelope, biogenesis and outer membrane (26 genes), while the 40 down-regulated genes were distributed among several COGs (Figure 4).

Hierarchical cluster analysis using Spotfire DecisionSite 8.2 was performed on normalized Cy3 (cDNA amplified from total RNA) signal intensity values of the microarray data from the four log phase and four stationary phase samples. All four samples from the log phase of growth clustered together, apart from those collected at stationary phase [see Additional file 3]. Moreover, genes that clustered together were indeed differentially expressed between the two growth conditions. The higher number of genes up-regulated in late-log growth phase coincides with a more active metabolism of late-log phase cultures compared to those at stationary phase.

In the following sections, we will focus our comments on those genes differentially expressed by microarray analysis that encode or are predicted to encode virulence factors, some of which may be involved in *Brucella*:host interaction.

### Protein-encoded genes which play a role in *Brucella *invasiveness in non-phagocytic cells did not have differential expression between the most and the least invasive cultures

Currently, only three *Brucella *gene products have been characterized as important for invasion in non-phagocytic cells. The *B. abortus *two-component regulatory system BvrR/BvrS encoded by *bvrR/bvrS *genes, regulates the structure of outer membrane components and plays a critical role in cell penetration and intracellular survival [11]. This two-component system is highly conserved in the genus *Brucella *[17], with ChvI/ChvG (encoded by BMEI2036 and BMEI2035, respectively) representing the *B. melitensis *homolog. In this study, neither of the two genes that encode this two-component system were differentially expressed between the most and the least invasive *B. melitensis *cultures.

Another *Brucella *invasive-characterized gene product is SP41, a surface protein that enables *B. suis *to attach and penetrate non-phagocytic cells [13]. The role of this gene has not been evaluated in *B. melitensis*, although a homolog is encoded by the *ugpB *gene present on the chromosome II of the *B. melitensis *16 M genome (BMEII0625). In this study, *ugpB *was not differentially expressed when global gene expression of *B. melitensis *cultures at late-log phase was compared to cultures at stationary growth phase.

Recently, a third gene product was reported to be involved in *Brucella *internalization in non-phagocytic cells [14]. In that study, a *B. melitensis *mutant with interruption in the BMEI0216 gene exhibited a marked decrease in its ability to invade HeLa cells at 1 and 2 h post-infection, suggesting the relevance of this gene in the *Brucella *invasion process after 1 h p.i. In this study, BMEI0216 was not found altered due to growth-phase.

Collectively, these results indicate that the higher invasiveness observed in *B. melitensis *cultures at late-log phase of growth under our experimental conditions was not due to the differential expression of these three characterized gene products. Also, these results suggest that genes encoding these three products were not growth-phase regulated in *B. melitensis *under our experimental conditions. However, they might be transcribed at a time that we did not measure, they could be constitutively expressed and act in concert with other factors, or they could be expressed following epithelial cell contact. It is perhaps worth noting that only one of these three gene products (hypothetical protein encoded by BMEI0216) has been effectively demonstrated to contribute to *B. melitensis *virulence, although after one hour post infection rather than the 30 minutes used in this study.

### Well-known *B. melitensis *virulence genes had different expression profiles in late-log phase of growth compared to stationary growth phase

Several genes whose products are known to be associated with *Brucella melitensis *virulence (although not yet demonstrated to influence in internalization by non-phagocytic cells), were differentially expressed between the most and the least invasive cultures. These included genes that encode T4SS proteins and the flagellar apparatus. The *virB *locus, for instances, encodes the Type IV Secretion System (T4SS) and plays a critical role in *Brucella *virulence and intracellular multiplication [18]. Three genes encoding components for the *virB *operon, such as *virB1 *(BMEII0025), *virB3 *(BMEII0027) and *virB10 *(BMEII0034) were up-regulated in *B. melitensis *cultures at late-log phase compared to stationary growth phase. Pathogenic bacteria produce flagella to promote colonization and invasion of mucosa. Brucellae are traditionally characterized as non-motile bacteria, yet the sequence of the *B. melitensis *genome contains three clusters of flagellar genes [19] and their participation in establishing chronic brucellosis has been established [20]. In our study, five genes such as *fliC *(BMEII0150), *fliF *(BMEII0151), *fliN *(BMEII1112), *flhA *(BMEII0166) and *flgD *(BMEII0164) which encode parts of the flagellar apparatus or regulate its expression, were differentially expressed in late-log phase cultures compared to stationary phase cultures. Previous studies reported scant influence of T4SS and flagella in the invasion process [20,21]. Thus, the highest penetration observed in late-log phase cultures was probably not due to the expression of these genes.

### Several transcriptional regulator genes were differentially expressed in late-log phase compared to stationary growth phase

Transcriptional regulators control bacterial gene expression in response to specific signals. Twenty-two genes encoding transcriptional regulators belonging to the AraC (BMEI1384, BMEII0143, BMEII0721), AsnC (BMEI1098, BMEI1845, BMEII0346), BetI (BMEI1379), DeoR (BMEII0426, BMEII0436, BMEII1093), GntR (BMEII0383, BMEII0807, BMEII1007), IclR (BMEI1717), LysR (BMEII0902, BMEII1077, BMEII1135), LuxR (BMEI1758), MarR (BMEII0520), MerR (BMEII0372, BMEII0467), and RpiR (BMEII0573) families were differentially expressed in late-log phase *B. melitensis *cultures compared to stationary phase cultures. Some of these transcription factors are known to be involved in positive regulation of gene expression (LuxR, AraC). Others are involved in repression (DeoR, MerR), while members of IclR and LysR families could be activators or repressors of gene expression [22]. Nevertheless, the contribution of these regulators and their targets to *B. melitensis *internalization epithelial cells has not been fully examined. The locus encoding the alternative sigma 32 factor (BMEI0280) that allows *Brucella *to survive under general stress situations was up-regulated in stationary phase cultures. The BMEI1789 locus that encodes a subunit of the other alternative sigma 54 factor (*rpoN*), which allows transcription of those genes involved in utilization of nitrogen and carbon sources and energy metabolism, was up-regulated in late-log phase cultures compared to stationary phase cultures.

Two-component transcriptional regulators are comprised of a cytoplasmic membrane-located sensor protein and a cytoplasmic response regulator protein [23]. Eight ORFs encoding for two-component response regulators have been identified in the *B. melitensis *16 M genome [19]. One of the signal transduction-encoded genes up-regulated in late-log phase cultures (*vsr*; BMEI1606), was previously identified in *B. melitensis *attenuated mutants [24]. The other (*hprK*; BMEI2034) is a central regulator of carbohydrate metabolism genes and also plays a role in virulence development of certain pathogens [25]. Although the molecular regulation of these response regulators in *B. melitensis *is currently unknown, understanding how *vsr*, *hprK *and others are regulated, could offer insight into *B. melitensis *virulence. Identifying the target genes of these transcriptional regulators would significantly clarify the role of growth-phase in *Brucella *physiology, metabolism and virulence regulation.

### Almost all differentially expressed genes encoding cell envelope and outer membrane components were up-regulated in late-log phase cultures

The ability of *Brucella *to invade cells has been linked to its outer membrane (OM) properties, as well as to structures built within the cell envelope [26,27]. Twenty-six genes directly involved in cell envelope and outer membrane biogenesis were differentially expressed at late-log compared to stationary phase of growth. These included genes that encode outer membrane proteins (BMEI0402, BMEI0786), lipoproteins (BMEI0991, BMEI1079), LPS (BMEI0418, BMEI0586, BMEI0833, BMEI1414), and peptidoglycan biosynthesis (BMEI0271, BMEI0576). The main COGs functional category of genes that were up-regulated in *B. melitensis *cultures at late-log compare to stationary phase of growth were ORFs encoding membrane transport proteins. These included genes encoding transporters specific for amino acids (BMEI0263–0264, BMEII0098–9 and BMEII0861 to II0864), carbohydrates (BMEI1580, BMEI1713, BMEII0621–2 and II0624) and uncharacterized transporters (BMEI1554, BMEII0481, BMEII0483, BMEII0662). Collectively, these data indicate an active conversion of metabolites to components of the cell envelope structure at late-log phase, which given the importance of the OM in virulence, could influence the initial *Brucella*:host cell interactions, facilitating attachment and entry into host cells.

## Discussion

The molecular mechanisms involved in the initial interactions between *Brucella *and epithelial cells have not been well characterized. Previous studies have used HeLa cells as a model for studying adhesion and internalization of *Brucella *spp. in non-professional phagocytic cells [9,10]. These studies found that brucellae bind to cellular receptors containing sialic acid residues and induce their own uptake by a local rearrangement of the host cell cytoskeleton around the invading organisms. The ability of the bacteria to adhere to and penetrate eukaryotic cells is a well orchestrated process that requires several factors/gene products in order to be successful [28]. To date, only a few *Brucella *gene products involved in non-phagocytic cell invasion have been identified [11,13,14]. This study was performed with the goal of better understanding initial molecular interactions between *Brucella *and its host through the molecular analysis of growth phase-specific gene regulation.

Our initial experiment indicated that cultures of *B. melitensis *at late-log growth phase in cell culture medium were more invasive to non-phagocytic cells than cultures at mid-log and stationary growth phases. Similar results have been observed for other invasive pathogens, such as *Salmonella *spp. or *Yersinia enterocolitica *[29,30]. Even with the high MOI used (1,000:1), *B. melitensis *were internalized in lower numbers by epithelioid-like HeLa cells at 30 min p.i. than reported in another study [14]. The difference in invasion may have been influenced by the F12K cell culture medium used to growth the agent. *B. melitensis *reach stationary phase at a lower OD (A600 nm) in F12K cell culture medium than in rich bacterial culture medium (Tryptic soy broth; TSB) or another cell culture medium (complete RPMI1640 medium supplemented with 10% HI-FBS) (0.72 vs. 1.6 vs. 0.95, respectively; data not shown). These results suggest that F12K medium apparently contains suboptimal nutrients for *Brucella *development. Even though, we grew *B. melitensis *in F12K medium and immediately added the bacteria to HeLa cells with the goal of reducing bacterial pre-infection manipulations (centrifugation, washes and transfer to fresh new media), which had probably modified the original transcriptome of the cultures, since bacterial gene expression changes quickly in response to environmental modification [31].

The relationship between growth phase and invasiveness is dependent upon the expression of bacterial virulence factors at different growth-phase. For instance, *Shigella flexneri *is more invasive during the early phase of their exponential growth, because invasion proteins (Ipa) are secreted in higher amounts during this growth phase [32]. *Salmonella enterica *and *Legionella pneumophila *have their secretion systems assembled and effector proteins properly stored in the cytoplasm only at the late exponential and stationary growth phases, respectively [28,33,34]. In order to understand why our system evoked greater invasiveness in *B. melitensis *cultures at late-log phase in the first 30 min p.i., we conducted a global gene expression detection study using cDNA microarray technology. Microarray analysis revealed that 454 genes were significantly differentially expressed between the most (late-log phase) and the least (stationary phase) invasive cultures [see Additional file 2]. As expected, the majority of the observed changes in gene expression were related to the bacterial response under the increased growth conditions in tissue culture media. For example, the up-regulation of genes associated with transcription and translation, nutrient metabolism, transport, and energy production and conversion all correspond to a more active metabolism of late-log phase cultures, compared to cultures at stationary phase. As was expected, several cell division- and DNA synthesis-related genes were also up-regulated at late-log phase, when the bacterial population was still actively growing. Alternatively, genes down-regulated in late-log phase were more heterogeneous in nature, demonstrating no predominant functional category. As expected, an increased expression of the locus BMEI0280 (*rpoH1*) encoding the alternative sigma 32 factor was observed in stationary phase cultures [35]. Sigma 32 factor regulates the transcription of heat shock genes, which allow the bacteria to survive not only an abrupt increase in temperature, but also general stress situations, such as nutrient limitations during stationary growth phase [36].

Previous work identified a role in *B. melitensis *invasion of HeLa cells for the hypothetical protein encoded by BMEI0216 ORF, which increases invasiveness only after 1 h p.i. [14]. That study clearly showed that the presence or absence of the gene transcript did not modify the ability of *B. melitensis *to invade HeLa cells during the first 30 min p.i., i.e. the *Brucella*-HeLa co-incubation time used in our study. Under our experimental conditions, BMEI0216 was not found phase growth regulated. These data suggest that BMEI0216 may be transcribed after prolonged host cell contact, thereby facilitating the invasion process at later time points. Further characterization of the regulation of this gene and its product is clearly warranted.

In seeking to identify possible contributors to the increased invasiveness of *B. melitensis *at late-log phase, the conversion of metabolites to components that alter cell envelope structure were evaluated. Altered outer membrane/cell wall topology would be expected to influence the initial bacteria:host cell interaction that may facilitate attachment and entry into host cells. In our study, a significant number of genes directly involved in cell envelope/outer membrane biogenesis were differentially expressed [see Additional file 2]. One of the genes up-regulated at late-log growth phase was the locus BMEI0402. The product of this gene has not yet been characterized in *B. melitensis*; however, it has high homology (63% sequence identity) to an immunogenic outer membrane protein, Omp31 (BMEII0844) [37]. Omp31 is a haemin-binding protein [38], which binds to, and extracts iron from, the host. Iron has been identified as a required element for epithelial invasion in microbial pathogens [39-41], and the expression of this locus, along with other iron-related genes in late-log phase cultures (BMEI0176–0177, BMEII0536, BMEII0567, BMEII0583, BMEII0704, BMEII0883, BMEII1120, BMEII1122), may influence the internalization ability of brucellae. SP41 is another surface-exposed outer membrane protein with a critical role in *Brucella suis *adherence to, and invasion of, non-phagocytic cells [13]. The role of this protein, which is encoded by the *ugpB *gene (BMEII0625) present in the chromosome II of *B. melitensis *16 M genome, was not previously described for *B. melitensis *adhesion to and/or penetration of epithelial cells. The transcript from the *ugpB *gene was not identified as differentially expressed in our cDNA microarray analysis between the most and the least invasive cultures. Therefore, under our experimental conditions, this OMP seems not to be involved in the higher invasiveness of the late-log phase cultures. It is possible that the composition of the cell culture medium does not induce the expression of *ugpB*, or it is also possible that *ugpB *is constitutively expressed and/or act in concert with other factors. Although genetic analysis reveals that *ugpB *may belong to an operon (BMEII0621 to II0625) that encodes for a sn-glycerol-3-phosphate ABC transporter [42], the experimental evidence does not support this hypothesis. A previous study showed that the product of *ugpB *in *B. suis *is indeed a surface-exposed protein with adhesion and invasion activity [13]. In fact, in this study, three of the transcripts predicted to encode the transport system [*ugpC *(BMEII00621) (ATP-binding thiprotein), *ugpE *(BMEII0622) and *ugpA *(BMEII0624) (permease proteins)] were highly up-regulated (> 50 fold) in late-log phase cultures, when compared to stationary phase cultures. In concordance with previous experimental evidence, our microarray data would support the finding of others that *ugpB *does not belong to an operon that encodes for a sn-glycerol-3-phosphate ABC transporter. In addition, our results support growth-phase regulation of the sn-glycerol-3-phosphate ABC transport system, which has been implicated in *Brucella *pathogenesis [24,43].

The ability of *Brucella *to invade host cells is linked to its OM properties. *B. melitensis *OMP profile changes during culture growth [44], as gene expression is transcriptional regulated by environmental conditions [12,45]. Although the different expression pattern observed in gene-encoding cell envelope products between late-log and stationary phase cultures here may have been due to medium composition, it is more likely that they are due to growth phase regulation. In this study, we did not evaluate the role of the OMP in internalization in epithelial cells and therefore their individual participation in increased invasiveness of late-log phase cultures could not be determined. Only two differentially expressed genes encoding for O-chain and peptidoglycan layer biosynthesis from this study [*perA *(BMEI1414) and *mtgA *(BMEI0271)], were previously evaluated in *Brucella *pathogenesis (extensively reviewed in [46]), although not in epithelial cells internalization [24,47]. Due to the importance that the cell envelope in initial host:pathogen interaction, the regulation and role of gene-encoding OM products differentially expressed in this study should be addressed in future studies.

Rapid adaptive physiological response to multiple environmental and cellular signals in bacteria is mainly mediated by transcriptional regulators and two-component regulatory systems. Prokaryotic genes putatively coding for transcriptional regulators are grouped in families based on sequence similarity and functional criteria. Twenty-two transcripts, belonging to 11 families of transcriptional regulators, were differentially expressed in our study [see Additional file 2]. It was recently reported that *B. melitensis *mutants for 12 of these 22 transcriptional regulators were not attenuated after one-week of infection in mice [48]. However, effects of these transcriptional regulators on internalization of *B. melitensis *by non-phagocytic cells have not been examined. Their contribution to invasion therefore remains unknown. LuxR is a well-known family of transcriptional activators that regulates various functions in microbes [49]. There are two loci (BMEI1758: *blxR *and BMEII1116: *vjbR*) that encode transcripts belonging to this family of transcriptional regulators in the *B. melitensis *genome, and their expression is required for transcription of virulence factors such as *virB *operon and flagella [50,51]. The transcriptional regulator *vjbR *was not differentially expressed in our study, but the other LuxR homolog (*blxR*), was 221-fold up-regulated in the late-log phase of growth, compared to stationary phase cultures. The targets of BlxR are currently unidentified, but regulatory effects on other transcriptional-regulatory proteins and proteins predicted to be involved in cell envelope biogenesis was observed [51]. It may be possible that some of these gene products regulated by BlxR positively influence *B. melitensis *invasion of HeLa cells. Analysis of the invasive phenotype of a *B. melitensis blxR *deletion mutant in HeLa cells would be the first step in determining the importance of this transcriptional regulator during the initial host:*Brucella *interaction, followed by the identification of the effector gene(s) it regulates. BvrR/BvrS is a well characterized two-component regulatory system that controls the expression of genes essential for *Brucella abortus *invasion to non-phagocytic cells [11,12]. High level of identity is present between *B. melitensis *ChvI/ChvG (encoded by BMEI2036 and BMEI2035, respectively) and the *B. abortus *BvrR/BvrS proteins [17]. In our study, no transcriptional change was observed in BMEI2036/I02035 ORFs between the most (late-log phase) and the least (stationary growth phase) invasive cultures. Likely, *Brucella *maintain a basal expression level of the regulatory locus, as a change in the phosphorylation of the protein required for activity rather than transcription.

Twenty ORFs dedicated to signal transduction were identified in *B. melitensis *genome [19]. The importance of some of them in *Brucella *virulence had been characterized lately, including *blxR*, *vjbR*, *ftcR *and *bvrR/bvrS *[12,45,50-52]. However, their contribution to internalization in non-phagocytic cells is less known. Recently, mutants with defective expression in two transcriptional regulators (*vjbR *and *bvrR/S*) had an altered pattern in initial host:pathogen interaction due to surface modifications [12,45]. Future identification of the target genes of these regulators would clarify *Brucella *physiology, metabolism and virulence regulation.

Several motility-related genes were more highly expressed at late-log phase compared to stationary phase, including kinesin-like protein, chemotaxis MotD protein and genes related to flagellum apparatus synthesis and functions, e.g. flagellin itself (96.6-fold). Flagellin has been well-characterized as a contributor to bacterial virulence through chemotaxis and adhesion to and invasion of host cells [53]. The extent to which flagellar machinery participates in the process of invasion seems to depend at least partly on the species of bacteria and/or the host cell type. For instance, flagellar-associated motility in *Salmonella *is not required but accelerates invasion of Caco-2 colonic epithelial cells [54], whereas the invasion of *Acanthamoeba astronyxis *by *Burkholderia pseudomallei *absolutely requires an intact flagellum apparatus [55]. In the case of *B. melitensis*, a previous study demonstrated that expression of flagella is growth curve-dependent and required for persistent disease in a mouse model but not for invasion in cellular models [20]. That study reported that a functional flagellum was assembled in early-log growth phase cultures but not at later time points. In our study, we did not analyze gene expression at early time points of the growth curve, but the results indicated that some flagellar genes were expressed more in late-log phase cultures as compared to stationary phase cultures. The differences in flagellum gene expression between the study of Fretin *et al*. (2005) and ours could be attributed to evaluation of different steps of the process (protein expression versus gene expression), different culture media used, or post-transcriptional regulatory mechanisms. The flagellar apparatus is built hierarchically under complex regulation. Thirty-one flagellar genes distributed in three clusters on chromosome II and along with three transcriptional regulators of flagellar system expression have been identified in *B. melitensis *[20,50-52]. However, the order of flagella gene expression and the whole system regulation in brucellae has not been established. Here, only five genes from two loci encoding different parts of the flagellar apparatus were differentially expressed in late-log phase cultures compared to stationary phase cultures. Detection of expression of some but not all genes from an operon is not uncommon with microarray data, due to the inherent nature of microarrays (e.g., simultaneous measurement of thousands of different transcripts, differences in hybridization kinetics, dye incorporation, etc) that produces variation that leads to some false negatives [56]. In a previous study, Rambow-Larsen *et al*. (2008) using a cDNA microarray, also identified only 5 of the 31 flagellar genes, belonging to different flagellar loci and encoding for distinct parts of the flagellar apparatus, expressed under a putative quorum-sensing regulator BlxR [51]. Similarly, microarray detected changes in expression of only some of the genes of the flagellar operon in *Salmonella enterica *serovar Typhimurium, which is transcribed with a polycistronic message, despite a 10-fold difference in some genes of each operon [57]. Two different functions, motility and protein secretion have been ascribed to flagella, but these roles have yet to be demonstrated in brucellae. We were not able to evaluate the role of *B. melitensis *flagellar gene expression in invasion under our experimental conditions, but undoubtedly, the presence of flagellar machinery and other adhesion/motility factors at late-log phase, and their exact contribution to the *Brucella *invasion process warrant further studies.

The *virB *operon has been reported to be essential for intracellular survival and multiplication of *Brucella *[21,58-60], but its role in adherence and internalization is contradictory [61,62]. In our study, three genes from the operon (*virB1*, *virB3 *and *virB10*) were up-regulated in late-log growth phase cultures compared to the stationary phase of growth. *virB *is transcribed as an operon, with no secondary promoters. It is maximally expressed in *B. melitensis *at the early exponential phase of the growth curve, and its expression decays as the bacteria reach the stationary phase [63]. However, the half-lives of the individual segments of the *virB *transcript are not known. Under our experimental conditions, it is possible that *virB *was expressed earlier in the growth curve, and the different rate of transcript degradation allowed the detection of expression of some genes of the operon in late-log phase but not in stationary phase cultures. Distinct half-lives of the individual segments of the polycistronic mRNA, results in the differential expression of each gene in the operon. This phenomenon has been well characterized in other bacteria [64,65], and is worthy to additional evaluation of *B. melitensis virB *operon. In addition, and similar to mention for flagellar genes, microarray could detect expression of some but not all genes from an operon, due to the inherent nature of the technique. Further, our analysis method was particularly stringent in order to greatly reduce false positives at the risk of additional false negatives. Thus, other genes in the *virB *operon were increased in expression such as *virB2*, *virB4*, *virB6*, *virB6 *and *virB11*, although not statistically significant because of the stringency of our statistical analysis.

Finally, genes with uncharacterized function that were differentially expressed at late-log phase compared with the stationary phase also deserve some special consideration. This group of "hidden genes" represents 22% of the differentially expressed genes identified in this study, and it may contain some of the heretofore unknown virulence factors utilized for *B. melitensis *to invade and infect the host, as was previously suggested [24,43,46]. Conversely, *Brucella *internalization should not be disregarded as a product of synergistic action among several gene products in non-phagocytic cells.

## Conclusion

Our study reveals that *B. melitensis *grown in cell culture medium at late-log phase are more invasive in non-phagocytic cells than cultures grown at mid-log or stationary growth phases. cDNA microarrays provide informative differential transcriptional profiles of the most (late-log growth phase) and the least (stationary growth phase) invasive *B. melitensis *cultures. We consider these data a platform for conducting further studies on the *Brucella*:host initial interaction. Since the roles of the majority of differentially expressed genes in this study are not well defined in *Brucella *pathogenesis, future studies on *Brucella *virulence can now be specifically focused to more precisely delineate the roles of candidate genes identified in this study.

## Methods

### Bacterial strains, media and culture conditions

Smooth virulent *Brucella melitensis *16 M Biotype 1 (ATCC 23456) (American Type Culture Collection, Manassas, VA), re-isolated from an aborted goat fetus, and its derivatives were maintained as frozen glycerol stocks. Individual 50 ml conical tubes were filled with 10 ml of cell culture medium [F12K medium (ATCC^®^) supplemented with 10% heat-inactivated fetal bovine serum (HI-FBS) (ATCC^®^)], inoculated with 0.1 ml (1:100 for mid-log cultures), 0.25 ml (1:40 for late-log phase cultures) and 1 ml (1:10 for stationary phase cultures) of a saturated culture of *B. melitensis *16 M and incubated overnight at 37°C with 5% CO_2_, loose lids and shaking (200 rpm). Growth curves of cultures were determined by comparing the optical density (OD) of the culture at 600 nm with bacterial colony forming units (CFU). Bacterial numbers were assessed by plating a serial dilution on tryptic soy agar (TSA) (BD, Franklin Lakes, NJ) and incubating at 37°C with 5% CO_2 _for 4 days.

### Determination of invasiveness

HeLa S3 cell line (ATCC CCL-2.2) between passages 8 and 15 was grown in F12K medium containing 10% HI-FBS at 37°C with 5% CO_2_. Twenty-four hours prior to infection, the cells were suspended and cultured in 25 cm^2 ^culture flasks (Corning, Corning, NY) at a concentration of 2 × 10^6 ^cells/flask and replaced in the incubator. Before infection, cells from 1 flask were detached and counted. For infection with *B. melitensis *16 M or its derivatives, the medium overlying the HeLa monolayers was replaced by a bacterial inoculum grown overnight in F12K cell culture media, at a multiplicity of infection of 1,000 bacteria per cell (MOI 1,000:1). Bacteria were centrifuged onto the cells at 800 × *g *for 10 min, followed by 30 min of incubation at 37°C with 5% CO_2_. Then, cells were washed once with phosphate buffer solution (PBS) to remove extracellular bacteria and subsequently re-incubated for 1 h in F12K media supplemented with 100 μg ml^-1 ^of gentamicin solution (Sigma, St. Louis, MO). To determine the viable number of intracellular bacteria, infected cultures were washed 3× with PBS and then lysed with 0.1% Triton X-100 (Sigma). Lysates were serially diluted and cultured on TSA plates for quantification of CFU.

### Isolation of total RNA from *B. melitensis *16 M

Total RNA was isolated by phenol-chloroform extraction from 4 different cultures of *B. melitensis *16 M grown in F12K supplemented with 10% HI-FBS at late-log and stationary growth phases, as previously described [66]. Briefly, ice-cold ethanol/phenol solution was added to the *B. melitensis *culture, and the bacteria were recovered by centrifugation. The media was then removed and the pellet suspended in TE buffer-lysozyme solution containing 10% SDS (Ambion, Austin, TX). After 2 min of incubation, acid water-saturated phenol (Ambion) was added to the lysate and mixed, and the sample was subsequently incubated for 6 min at 64°C. Tubes were kept on ice for at least 2 min and then centrifuged at maximum speed. The upper layer, containing the RNA, was transferred to a new tube, mixed with an equal volume of chloroform (Sigma) and then separated by centrifugation. The aqueous phase was mixed with 100% cold ethanol and stored at -20°C. After at least one hour of incubation, RNA was pelleted by centrifugation, washed in 80% ethanol and suspended in DEPC-treated water (Ambion) containing 2% DTT and 1% RNase inhibitor (Promega, Madison, WI). Contaminant genomic DNA was removed by RNase-free DNase I treatment (Ambion) according to the manufacturer's instructions, and samples were stored at -80°C until used. RNA concentration was quantified using the NanoDrop^® ^ND-1000 (NanoDrop, Wilmington, DE), and quality was determined using the Agilent 2100 Bioanalyzer (Agilent, Palo Alto, CA).

### Isolation and labeling of *B. melitensis *genomic DNA

A pellet from a saturated culture of *B. melitensis *16 M grown in tryptic soy broth (TSB) (BD) was washed with 25 ml of J-buffer [0.1 M Tris pH 8.0; 0.1 M EDTA; 0.15 M NaCl] and then lysed in 1 ml of J-buffer containing 10% lysozyme solution (10 mg/ml in 0.25 M Tris, pH 8.0). After 10 min of incubation, DNA was released from the cells by sodium N-lauroyl sarcosine (Sigma) treatment followed by degradation of RNA by DNase-free RNase (Roche Applied Science, Indianapolis, IN) treatment and digestion of proteins with proteinase K (Roche Applied Science). The resulting solution was transferred to a dialysis bag and dialyzed against TE [10 mM Tris, pH 8.0 and 1 mM EDTA] overnight at 37°C. DNA was subsequently extracted twice using neutral water-saturated phenol (Ambion) first and then ether (Sigma) before dialyzing overnight against TE. DNA concentration was quantified by NanoDrop^® ^ND-1000 (NanoDrop) and stored at 4°C until used.

*B. melitensis *genomic DNA was labeled overnight by directed incorporation of Cy5-dCTP (Amersham Pharmacia Biosciences, Piscataway, NJ) using random primers solution and Klenow fragment from the BioPrime DNA labeling system kit (Invitrogen, Carlsbad, CA) and 50× dNTPs (1:2 dCTP) (Invitrogen). The reaction was stopped by adding 5 μl of stop buffer from the BioPrime kit, and unincorporated Cy5 dye was removed using a PCR purification kit (Qiagen, Valencia, CA). The labeled DNA was eluted in 1 mM Tris pH 8.0 and kept in the dark at 4°C until used.

### Construction of cDNA microarrays

A set of unique 70-base oligonucleotides representing 3,227 ORFs of *B. melitensis *strain 16 M plus unique/divergent genes from *B. abortus *and *B. suis *were designed and purchased from Sigma-Genosys (The Woodland, TX). Oligonucleotides were suspended in 3× SSC (Ambion) at a final concentration of 40 μM before robotic arrayed in triplicate onto ultraGAPS coated glass slides (Corning) using a Spotarray 72 microarray printer (Perkin Elmer, Downer's Grove, ILL). Printed slides were steamed, UV cross-linked and stored in desiccators until use.

### Sample preparation and slide hybridization

Labeling and hybridization procedures were adapted from a protocol developed by The Institute for Genomic Research [67]. Briefly, 10 μg of *B. melitensis *16 M total RNA were reverse-transcribed overnight using 6 mg of random hexamer primers (Invitrogen), 0.6 μl 50× dNTPs (Invitrogen)/aa-dUTP (Ambion) mix (2:3 aa-dUTP:dTTP) and 400 U Superscript III (Invitrogen). The reaction was stopped by incubating the samples with 1 M NaOH at 65°C for 15 min and neutralized by subsequently adding 1 M HCl. Unincorparated aa-dUTPs and free amines were removed by column passage (Qiagen PCR Purification Kit, Quiagen). Speedvac-dried samples were rehydrated in 0.1 M Na_2_CO_3 _buffer (pH 9.0) and labeled with Cy3-ester (Amersham Pharmacia Biosciences). After one hour incubation in the dark, uncoupled dye was removed by column filtration (Qiagen) and Cy3 incorporation calculated using the NanoDrop^® ^ND-1000 (NanoDrop). Dry labeled cDNA samples were re-suspended in nuclease-free water (Ambion) and mixed with 0.5 μg of labeled gDNA to a final volume of 35 μl. Samples were heated at 95°C for 5 min and then kept at 45°C until hybridization, at which point 35 μl of 2× formamide-based hybridization buffer [50% formamide; 10× SSC; 0.2% SDS] was added to each sample. Samples were then well-mixed and applied to custom 3.2 K *B. melitensis *oligo-arrays. Four slides for each condition (i.e. late-log and stationary growth phases) were hybridized at 45°C for ~ 20 h in a dark, humid chamber (Corning) and then washed for 10 min at 45°C with low stringency buffer [1× SSC, 0.2% SDS], followed by two 5-min washes in a higher stringency buffer [0.1× SSC, 0.2% SDS and 0.1× SSC] at room temperature with agitation. Slides were dried by centrifugation at 800 × *g *for 2 min and immediately scanned. Prior to hybridization, oligo-arrays were pretreated by washing in 0.2% SDS, followed by 3 washes in distilled water, and immersed in pre-hybridization buffer [5× SSC, 0.1% SDS; 1% BSA in 100 ml of water] at 45°C for at least 45 min. Immediately before hybridization, the slides were washed 4× in distilled water, dipped in 100% isopropanol for 10 sec and dried by centrifugation at 1,000 × *g *for 2 min.

### Data acquisition and microarray data analysis

Immediately after washing, the slides were scanned using a commercial laser scanner (GenePix 4100; Axon Instruments Inc., Foster City, CA). The genes represented on the arrays were adjusted for background and normalized to internal controls using image analysis software (GenePixPro 4.0; Axon Instruments Inc.). Genes with fluorescent signal values below background were disregarded in all analyses. Data were analyzed using GeneSpring 7.0 (Silicon Genetics, Redwood City, CA), Significance Analysis of Microarrays (SAM) (Stanford University, Stanford, CA) and Spotfire DecisionSite 8.2 (Spotfire, Inc., Somerville, MA). Computational hierarchical cluster analysis and analysis of variance (ANOVA) were performed using Spotfire DecisionSite 8.2. ANOVA was also performed, as an additional filtering aid, using GeneSpring. For each software program used, data were first normalized by either mean (for Spotfire pairwise comparisons and SAM two-class comparisons) or percentile value (for GeneSpring analyses). Normalizations against genomic DNA were performed as previously described [15]. Microarray data have been deposited in Gene Expression Omnibus (GEO) database at NCBI [Accession # GSE11192].

### Validation of microarray results

One randomly selected gene from every Clusters of Orthologous Groups of proteins (COGs) functional category (n = 18) that was differentially expressed between late-log and stationary growth phases based on microarray results, was analyzed by quantitative RT-PCR (qRT-PCR). Two micrograms from the same RNA samples used for microarray hybridization were reverse-transcribed using TaqMan^® ^(Applied Biosystems, Foster City, CA). For relative quantification of target cDNA, samples were analyzed in individual tubes in the SmartCycler II (Cepheid, Sunnyvale, CA). One SmartMix bead (Cepheid) was used for each 2 – 25 μl PCR reaction along with 20 ng of cDNA, 0.2× SYBR Green I dye (Invitrogen) and 0.3 μM forward and reverse primers (Sigma Genosys) designed using Primer Express Software v2.0 (Applied Biosystems) [see Additional file 4] to produce an amplicon length of about 150 bp. For each gene tested, the individual calculated threshold cycles (Ct) in late-log and stationary phase samples were averaged among each condition and normalized to the Ct of the *B. melitensis *16S rRNA (*rrnA*) gene from the same cDNA samples before calculating the fold change using the ΔΔC_t _method (Applied Biosystems Prism SDS 7700 User Bulletin #2). For each primer pair, a negative control (water) and an RNA sample without reverse transcriptase (to determine genomic DNA contamination) were included as controls during cDNA quantification. All samples were run on a 1% agarose gel after qRT-PCR to verify that only a single band was produced. Array data were considered valid if the fold change of each gene tested by qRT-PCR was > 2.0 and in the same direction as determined by microarray analysis.

### Statistical analysis

Three independent experiments were performed to determine the invasiveness of cultures of *B. melitensis *16 M at different phases of growth. Statistical significance was determined using Student's *t *test, with a *P *value < 0.05 considered as significant.

## Authors' contributions

CAR conceived, designed and performed the experiments, and drafted the manuscript. CLG performed the computational analysis and drafted the manuscript. SDL conceived and designed the experiments and critically revised the manuscript. HRG helped to analyze the data and critically revised the manuscript. LGA conceived and coordinated the study and helped to draft the manuscript. All authors read and approved the final manuscript.

## Acknowledgements

We thank Dr. Tomas A. Ficht for providing the *B. melitensis *16 M strain, Dr. Renée M. Tsolis for critical reading of the manuscript and the anonymous reviewers for their helpful comments to improve the quality of the manuscript. We are grateful to the Western Regional Center of Excellence (WRCE) Pathogen Expression Core (Dr. John Lawson, Dr. Mitchell McGee, Dr. Rhonda Friedberg and Dr. Stephen A. Johnston, A.S.U.) for developing and printing the *B. melitensis *cDNA microarrays. L.G.A. and H.R.G were supported by grants from the NIH/NIAID Western Regional Center of Excellence 1U54 AI057156-01. L.G.A is also supported by the U.S. Department of Homeland Security National Center of Excellence for Foreign Animal and Zoonotic Disease Defense ONR-N00014-04-1-0 grant. C.A.R. was supported by I.N.T.A.-Fulbright Argentina Fellowship. C.L.G. received support from an NIH cardiology fellowship, Cardiology Department, University of Texas Southwestern Medical Center.

## Supplementary Material

Additional file 1 **Fluorescent signal values of *B*. *melitensis *gDNA in microarrays co-hybridized with *B. melitensis *RNA at late-log and stationary growth phases**. Average Cy5 (gDNA) fluorescent signal values for *B*. *melitensis *grown in F12K tissue culture medium to late-log and stationary phases (4 arrays each) were plotted in Excel. Each dot represents the signal value for an individual spot on the array. Fluorescent signal values for gDNA co-hybridized with *B. melitensis *RNA extracted at stationary growth phase are indicated on the ordinate, and fluorescent signal values for gDNA co-hybridized with *B. melitensis *RNA extracted at late-log phase are on the abscissa. Stat refers to stationary phase, log refers to late-log phase, and gDNA refers to genomic DNA. The R-squared value (0.8841) is displayed in the upper right-hand quadrant of the graph. Click here for file

Additional file 2 **Table A.1**. Genes significantly altered in *B*. *melitensis *grown in F12K tissue culture medium to late-log phase, compared to stationary phase under the same conditions. Click here for file

Additional file 3 **Hierarchical cluster of genes from *B*. *melitensis *grown to stationary and late-log phases**. Hierarchical clustering was performed on normalized Cy3 (transcript) signal intensity values from 8 arrays using Spotfire DecisionSite 8.2 software. Columns represent samples, and rows represent individual probes/genes. Higher signal values are shown in red, and lower signal values are shown in green. Note that all four stationary phase samples clustered together and apart from all four log phase cultures (tick line indicates individual growth phase replicate). Numbers in the top left of the figure indicate the number of cluster levels. The number below (-0.913) represents the calculated similarity measure between the two subnodes in each node. Click here for file

Additional file 4 **RT-PCR primers**. The table describes the primers used for testing *B. melitensis *gene expression by Real time – PCR. Click here for file
