# Supplementary figures and images for: Brucella melitensis global gene expression study provides novel information on growth phase-specific gene regulation with potential insights for understanding Brucella:host initial interactions
Source: BMC Microbiol. 2009 May 6;9:81. doi: 10.1186/1471-2180-9-81 (PMC2684542; doi:10.1186/1471-2180-9-81)

**Additional file 1**

**
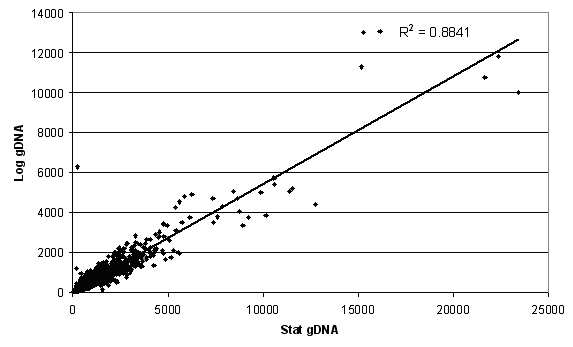
**

Supplement: Additional file 1 — Fluorescent signal values of B. melitensis gDNA in microarrays co-hybridized with B. melitensis RNA at late-log and stationary growth phases. Average Cy5 (gDNA) fluorescent signal values for B. melitensis grown in F12K tissue culture medium to late-log and stationary phases (4 arrays each) were plotted in Excel. Each dot represents the signal value for an individual spot on the array. Fluorescent signal values for gDNA co-hybridized with B. melitensis RNA extracted at stationary growth phase are indicated on the ordinate, and fluorescent signal values for gDNA co-hybridized with B. melitensis RNA extracted at late-log phase are on the abscissa. Stat refers to stationary phase, log refers to late-log phase, and gDNA refers to genomic DNA. The R-squared value (0.8841) is displayed in the upper right-hand quadrant of the graph. [file 1471-2180-9-81-S1.doc]
