# Supplementary material for: Brucella melitensis global gene expression study provides novel information on growth phase-specific gene regulation with potential insights for understanding Brucella:host initial interactions
Source: BMC Microbiol. 2009 May 6;9:81. doi: 10.1186/1471-2180-9-81 (PMC2684542; doi:10.1186/1471-2180-9-81)
Supplement: Additional file 2 — Table A.1. Genes significantly altered in B. melitensis grown in F12K tissue culture medium to late-log phase, compared to stationary phase under the same conditions. [file 1471-2180-9-81-S2.doc]

**Additional file 2. Table A.1.** Genes significantly altered in *B*. *melitensis* grown in F12K tissue culture medium to late-log phase, compared to stationary phase under the same conditions.

| **Locus ID** | **Gene product** | **SAM** | **Spotfire** | **GSpring** | **ANOVA** | |
| --- | --- | --- | --- | --- | --- | --- |
|  |  |  | **FC** |  | **(*P*)** | |
|  | | | | | | |
| **DNA replication, recombination and repair** | | | | | | |
| BMEI0453 | MutT/NUDIX family protein | 5.2 | 69.1 | 60.8 | | 0.007082 |
| BMEI0462 | Phosphohydrolase (MutT/NUDIX family protein) | 37.8 | 122.2 | 132.0 | | 0.01818 |
| BMEI0880 | Single-strand binding protein | 2.9 | 124.0 | 124.9 | | 0.029169 |
| BMEI0901 | Resolvase | 2.5 | 2.8 | 2.8 | | 0.005101 |
| BMEI1321 | DNA polymerase, bacteriophage-type | 3.1 | 3.8 | 3.9 | | 0.008698 |
| BMEI1362 | Chromosomal replication initiator protein DnaA | 3.8 | 300.9 | 449.2 | | 0.029257 |
| BMEI1409 | Transposase | 11.7 | 201.6 | 264.4 | | 0.013443 |
| BMEI1420 | Transposase | 123.0 | 123.0 | 123.0 | | 0.009518 |
| BMEI1422 | Transposase | 75.6 | 94.8 | 95.0 | | 0.005182 |
| BMEI1485 | Replicative DNA helicase | 15.2 | 66.6 | 60.5 | | 0.024788 |
| BMEI1815 | Transposase | 6.2 | 80.0 | 70.3 | | 0.037454 |
| BMEI1910 | Recombination protein RecR | 4.3 | 88.2 | 77.7 | | 0.028354 |
| BMEI1942 | DNA polymerase III subunit beta | 5.95 | 160.0 | 170.9 | | 0.040039 |
| BMEII0183 | Transposase | 5.8 | 8.7 | 9.8 | | 0.011806 |
| BMEII0290 | DNA polymerase III subunit epsilon | 4.0 | 55.4 | 76.8 | | 0.027906 |
| BMEII0447 | Transporter | 2.5 | 4.9 | 5.3 | | 0.00539 |
| BMEII0527 | Exodeoxyribonuclease VII large subunit | 4.0 | 112.5 | 111.0 | | 0.001261 |
| BMEII0663 | Phosphohydrolase (MutT/NUDIX family protein) | 98.8 | 141.2 | 152.2 | | 0.025194 |
| BMEII0676 | DNA topoisomerase IV subunit B | 2.53 | 2.8 | 2.9 | | 0.014043 |
| BMEII0713 | Transposase | 174.2 | 311.0 | 324.6 | | 0.000368 |
| BMEII0716 | Transposase | 6.4 | 67.6 | 63.1 | | 0.041021 |
|  |  |  |  |  | |  |
| **Transcription** | | | | | | |
| BMEI0280 | RNA polymerase sigma 32 factor | -5.0 | -10.0 | -4.3 | | 0.004316 |
| BMEI0510 | Leucine-responsive regulatory protein | 59.4 | 87.2 | 76.0 | | 0.004841 |
| BMEI1098 | Transcriptional regulatory protein, AsnC family | 4.3 | 10.7 | 10.1 | | 0.032585 |
| BMEI1379 | Transcriptional regulator BetI | 6.0 | 155.2 | 169.8 | | 0.008584 |
| BMEI1384 | Transcriptional regulator, AraC family | -20.0 | -20.7 | -17.0 | | 0.005425 |
| BMEI1717 | Transcriptional regulator, IclR | 4.3 | 12.2 | 13.3 | | 0.006167 |
| BMEI1758 | Transcriptional activator, LuxR family, *blxR* | 221.6 | 221.6 | 221.6 | | 0.011148 |
| BMEI1789 | DNA-directed RNA polymerase subunit N (sigma-54 factor rpon) (*rpoN*) | 7.56 | 113.6 | 92.1 | | 0.017962 |
| BMEI1845 | Transcriptional regulatory protein, AsnC family | 100.0 | 100.0 | 100.0 | | 0.018725 |
| BMEII0092 | Replication protein B | 54.4 | 74.6 | 71.5 | | 0.017128 |
| BMEII0127 | Acetate operon repressor | 2.6 | 34.9 | 41.4 | | 0.018305 |
| **TABLE A.1. (continued)** | | | | | | |
|  |  |  |  |  | |  |
| **Locus ID** | **Gene product** | **SAM** | **Spotfire** | **GSpring** | | **ANOVA** |
|  |  |  | **FC** |  | | **(*P*)** |
|  |  |  |  |  | |  |
| BMEII0143 | Transcriptional regulator, AraC family | 13.1 | 120.6 | 110.8 | | 0.011801 |
| BMEII0346 | Transcriptional regulatory protein, AsnC family | 39.3 | 39.1 | 39.1 | | 0.00386 |
| BMEII0372 | Transcriptional regulator, MerR family | 3.9 | 91.5 | 64.7 | | 0.039435 |
| BMEII0383 | Transcriptional regulator, GntR family | 37.4 | 37.4 | 37.4 | | 0.026822 |
| BMEII0426 | Transcriptional regulator, DeoR family | 44.9 | 44.9 | 44.9 | | 0.005492 |
| BMEII0436 | Transcriptional regulator, DeoR family | 8.8 | 94.1 | 98.6 | | 2.60E-03 |
| BMEII0467 | Transcriptional regulator, MerR family | 37.0 | 88.1 | 86.1 | | 0.021261 |
| BMEII0520 | Transcriptional regulator, MarR family | -7.2 | -7.4 | -6.4 | | 0.009266 |
| BMEII0573 | Transcriptional regulator, RpiR family | 56.5 | 103.4 | 109.5 | | 0.026452 |
| BMEII0721 | Transcriptional regulator, AraC family | 13.7 | 107.2 | 98.6 | | 0.013143 |
| BMEII0807 | Transcriptional regulator, GntR family | 4.4 | 219.3 | 202.1 | | 0.007073 |
| BMEII0902 | Transcriptional regulator protein, LysR family | 34.2 | 34.2 | 34.2 | | 0.007487 |
| BMEII1007 | Transcriptional regulator, GntR family | 7.0 | 61.0 | 508.9 | | 0.001131 |
| BMEII1077 | Transcriptional regulator protein, LysR family | 3.2 | 55.4 | 72.3 | | 0.0216 |
| BMEII1093 | Glycerol-3-phosphate regulon repressor (DeoR Family) | 386.5 | 386.5 | 386.5 | | 0.004118 |
| BMEII1135 | Transcriptional regulator protein, LysR family | 2.3 | 3.1 | 3.1 | | 0.008648 |
|  |  |  |  |  | |  |
| **Translation, ribosomal structure and biogenesis** | | | | | | |
| BMEI0277 | Heat shock protein 15 | 2.4 | 3.3 | 3.2 | | 0.008141 |
| BMEI0616 | tRNA delta(2)-isopentenylpyrophosphate transferase | 9.4 | 73.7 | 69.1 | | 0.005714 |
| BMEI0890 | Queuine tRNA-ribosyltransferase | 5.8 | 174.1 | 212.4 | | 0.011321 |
| BMEI0934 | ATP-dependent RNA helicase RhlE | 3.4 | 46.4 | 36.4 | | 0.032457 |
| BMEI1671 | Translation initiation factor IF-1 | 2.4 | 4.3 | 4.8 | | 0.031672 |
| BMEI1798 | 23S ribosomal RNA methyltransferase | 164.3 | 171.4 | 172.6 | | 0.005135 |
| BMEI1959 | Methyltransferase | 213.5 | 213.5 | 213.5 | | 0.004073 |
| BMEI1960 | Methyltransferase | 8.1 | 85.8 | 89.3 | | 0.021941 |
| BMEII0276 | Ribonuclease P | 6.4 | 274.5 | 226.0 | | 0.008062 |
| BMEII0278 | Translation initiation inhibitor | 4.4 | 8.1 | 7.2 | | 0.004336 |
| BMEII0597 | 23S ribosomal RNA methyltransferase | 4.4 | 7.8 | 8.4 | | 0.012347 |
| BMEII0890 | 16S ribosomal RNA m(5)C 967 methyltransferase | 30.7 | 88.8 | 77.7 | | 0.02906 |
|  |  |  |  |  | |  |
| **Nucleotide metabolism** | | | | | | |
| BMEI0358 | Deoxyuridine 5'-triphosphate nucleotidohydrolase | 6.95 | 114.4 | 100.3 | | 3.90E-05 |
| BMEI0608 | Thymidylate synthase | 91.7 | 91.7 | 91.7 | | 0.012037 |
| BMEI1281 | Dihydroorotase | 2.3 | 2.5 | 2.5 | | 0.002812 |
| BMEI1571 | Guanine deaminase | -5.1 | -11.4 | -4.9 | | 0.011304 |
| BMEI1575 | Xanthine dehydrogenase | 2.9 | 76.7 | 79.6 | | 0.021288 |
| BMEI1576 | Xanthine dehydrogenase | 68.3 | 257.3 | 254.9 | | 0.000524 |
| BMEI1639 | Dihydropyrimidine dehydrogenase | 94.7 | 123.0 | 122.3 | | 0.014601 |
| BMEII0088 | Inosine-uridine preferring nucleoside hydrolase | 10.5 | 785.5 | 1058.8 | | 0.021628 |
| BMEII0627 | Probable adenine deaminase | 3.4 | 177.2 | 223.8 | | 0.013583 |
|  |  |  |  |  | |  |
| **TABLE A.1. (continued)** | | | | | | |
|  |  |  |  |  | |  |
| **Locus ID** | **Gene product** | **SAM** | **Spotfire** | **GSpring** | | **ANOVA** |
|  |  |  | **FC** |  | | **(*P*)** |
|  |  |  |  |  | |  |
| **Carbohydrate metabolism** | | | | | | |
| BMEI0344 | Phosphoglucosamine mutase/phosphoacetylglucosamine mutase/phosphomannomutase | 51.3 | 84.9 | 76.9 | | 0.010824 |
| BMEI0921 | UDP-glucose 4-epimerase | -7.6 | -11.6 | -6.7 | | 0.005944 |
| BMEI0974 | Ribose-5-phosphate isomerase A | 97.5 | 158.0 | 157.0 | | 0.008367 |
| BMEI1237 | UDP-glucose 4-epimerase | 2.6 | 2.6 | 2.8 | | 0.039446 |
| BMEI1436 | Pyruvate phosphate dikinase | 5.5 | 67.3 | 58.8 | | 0.029235 |
| BMEI1570 | Putative hydroxypyruvate reductase | 3.7 | 116.9 | 114.9 | | 0.040269 |
| BMEII0355 | D-galactose 1-dehydrogenase | 4.3 | 85.1 | 111.2 | | 0.020829 |
| BMEII0430 | Erythritol kinase | 3.5 | 103.2 | 118.8 | | 0.03274 |
| BMEII0476 | Uronate isomerase | 55.8 | 55.8 | 55.8 | | 0.017535 |
| BMEII0568 | Myo-inositol-1(or 4)-monophosphatase | 2.7 | 4.7 | 4.6 | | 0.023251 |
| BMEII0574 | Myo-inositol 2-dehydrogenase | 154.3 | 154.3 | 154.3 | | 0.023522 |
| BMEII0724 | Endoglucanase H | 3.7 | 208.4 | 206.4 | | 0.013526 |
| BMEII0823 | Glycerol kinase | 58.9 | 58.9 | 58.9 | | 0.005347 |
| BMEII0850 | GDP-fucose synthetase | 6.5 | 285.5 | 453.3 | | 0.032125 |
| BMEII1095 | L-fuculose phosphate aldolase | 5.5 | 164.6 | 194.1 | | 0.010794 |
|  |  |  |  |  | |  |
| **Lipid metabolism** | | | | | | |
| BMEI0099 | 3-hydroxybutyryl-CoA dehydrogenase | 3.1 | 10.9 | 11.5 | | 0.03388 |
| BMEI0166 | Acyl-CoA thioesterase II | 3.85 | 76.7 | 81.4 | | 0.032527 |
| BMEI0477 | Monoamine oxidase regulatory protein, putative | 41.3 | 64.1 | 63.4 | | 0.035528 |
| BMEI0688 | 3-hydroxyisobutyrate dehydrogenase | 81.9 | 82.0 | 81.9 | | 0.020739 |
| BMEI0897 | Glutaryl-CoA dehydrogenase | 2.7 | 68.1 | 66.2 | | 0.021585 |
| BMEI1024 | 3-hydroxyisobutyrate dehydrogenase | 8.8 | 177.8 | 185.3 | | 0.018569 |
| BMEI1235 | Short-chain dehydrogenase | 49.2 | 49.2 | 49.2 | | 0.010489 |
| BMEI1473 | 3-oxoacyl-(acyl carrier protein) synthase | 2.3 | 2.4 | 2.4 | | 0.003662 |
| BMEI1478 | Acyl-carrier-protein S-malonyltransferase | 9.1 | 62.1 | 49.2 | | 0.023269 |
| BMEI1709 | Oxidoreductase UcpA | 2.7 | 3.8 | 3.4 | | 0.028269 |
| BMEI1861 | Arylesterase precursor | 5.2 | 134.7 | 154.4 | | 0.029325 |
| BMEII0047 | Lysophospholipase L2 | 47.2 | 47.2 | 47.2 | | 0.008023 |
| BMEII0239 | Cardiolipin synthetase | 14.4 | 170.8 | 182.4 | | 0.017839 |
| BMEII0514 | 3-ketoacyl (acyl-carrier-protein) reductase | 4.5 | 139.2 | 197.5 | | 0.033898 |
| BMEII1103 | Phosphatidylglycerophosphatase B | 3.6 | 148.1 | 143.9 | | 0.02058 |
|  |  |  |  |  | |  |
| **Amino acid metabolism** | | | | | | |
| BMEI0028 | L-sorbose dehydrogenase [FAD] | 13.1 | 76.2 | 63.6 | | 0.027971 |
| BMEI0559 | 5,10-methylenetetrahydrofolate reductase | 2.7 | 3.0 | 2.9 | | 0.017735 |
| BMEI0591 | Xaa-Pro aminopeptidase | 94.4 | 144.8 | 134.7 | | 0.01548 |
| BMEI0617 | Acetolactate synthase III large subunit | 54.4 | 146.4 | 125.8 | | 0.014389 |
| BMEI0647 | Urease alpha subunit | 42.7 | 42.7 | 42.7 | | 0.021861 |
| BMEI0706 | CobC protein | 46.8 | 46.8 | 46.8 | | 0.016867 |
| **TABLE A.1. (continued)** | | | | | | |
|  |  |  |  |  | |  |
| **Locus ID** | **Gene product** | **SAM** | **Spotfire** | **GSpring** | | **ANOVA** |
|  |  |  | **FC** |  | | **(*P*)** |
|  |  |  |  |  | |  |
| BMEI0730 | Lactoylglutathione lyase | 163.9 | 163.9 | 163.9 | | 0.011144 |
| BMEI1365 | Protease II | 88.2 | 260.5 | 308.2 | | 0.015829 |
| BMEI1380 | Choline dehydrogenase | 6.2 | 295.9 | 360.4 | | 0.025683 |
| BMEI1617 | O-succinylhomoserine sulfhydrylase | -18.6 | -136.3 | -14.6 | | 0.006793 |
| BMEI1719 | Sarcosine oxidase gamma subunit | 4.0 | 27.3 | 10.5 | | 0.005638 |
| BMEI1720 | Sarcosine oxidase alpha subunit | 24.0 | 64.0 | 64.1 | | 0.021308 |
| BMEI1781 | Carbamoyl-phosphate synthase large chain | 22.8 | 124.4 | 148.7 | | 0.009883 |
| BMEI1905 | Prephenate dehydratase | 4.7 | 154.1 | 118.3 | | 0.033418 |
| BMEII0012 | Oligoendopeptidase F | 41.9 | 96.1 | 100.6 | | 0.021343 |
| BMEII0040 | Glutamate synthase [NADPH] large chain | 14.8 | 92.1 | 90.6 | | 0.024754 |
| BMEII0049 | N-formylglutamate deformylase | 8.6 | 69.6 | 73.0 | | 0.020268 |
| BMEII0134 | 5-carboxymethyl-2-hydroxymuconate delta-isomerase | 234.53 | 298.8 | 302.4 | | 0.002041 |
| BMEII0135 | 5-carboxymethyl-2-hydroxymuconate semialdehyde dehydrogenase | 3.16 | 9.7 | 10.3 | | 0.037007 |
| BMEII0136 | Homoprotocatechuate 2,3-dioxygenase | 23.7 | 170.7 | 164.3 | | 0.026465 |
| BMEII0404 | 3-isopropylmalate dehydrogenase | 5.0 | 103.4 | 89.7 | | 0.01957 |
| BMEII0546 | Metal-activated pyridoxal enzyme | 2.5 | 2.6 | 2.6 | | 0.004646 |
| BMEII0582 | Sarcosine oxidase beta subunit | 77.9 | 147.5 | 151.7 | | 0.002203 |
| BMEII0756 | N-acetylglucosamine kinase | 13.7 | 147.8 | 107.9 | | 0.018282 |
| BMEII0907 | Glutaminase | 4.6 | 152.4 | 128.0 | | 0.03523 |
| BMEII0910 | Glutamate decarboxylase beta | 3.1 | 136.3 | 113.9 | | 0.041125 |
| BMEII0964 | Asparagine synthetase B (glutamine-hydrolyzing) | 14.4 | 331.3 | 323.2 | | 0.00443 |
| BMEII1054 | ATP phosphoribosyltransferase | 2.3 | 2.4 | 2.3 | | 0.002473 |
| BMEII1055 | ATP phosphoribosyltransferase regulatory subunit | 5.8 | 251.7 | 103.8 | | 0.038745 |
|  |  |  |  |  | |  |
| **Secondary metabolite biosynthesis, transport and metabolism** | | | | | | |
| BMEI1560 | Salicylaldehyde dehydrogenase | 3.9 | 81.9 | 68.3 | | 0.023819 |
| BMEI1860 | Hypothetical transmembrane oxidoreductase | 2.4 | 2.7 | 2.5 | | 0.018287 |
| BMEII0078 | 2,3-dihydroxybenzoate-AMP ligase | 221.0 | 221.0 | 221.0 | | 0.015707 |
| BMEII0079 | Isochorismatase | 55.6 | 105.4 | 92.1 | | 0.010724 |
| BMEII0580 | Probable blue-copper protein YacK precursor | 2.7 | 6.8 | 7.7 | | 0.019374 |
|  |  |  |  |  | |  |
| **Energy production and conversion** | | | | | | |
| BMEI0475 | Cytochrome C1 | 15.13 | 180.3 | 164.8 | | 0.016951 |
| BMEI0548 | Flavohemoprotein | 99.2 | 99.2 | 99.2 | | 0.004279 |
| BMEI0836 | Citrate synthase | -3.7 | -5.0 | -3.3 | | 0.008278 |
| BMEI0898 | Predicted acyl-CoA transferase/Carnitine dehydratase | 2.8 | 3.7 | 4.1 | | 0.017818 |
| BMEI1462 | Cytochrome C oxidase polypeptide III | 5.6 | 152.0 | 114.1 | | 0.008024 |
| BMEI1465 | Cytochrome C oxidase polypeptide I | 2.85 | 3.3 | 3.2 | | 0.0006 |
| BMEI1591 | Ferredoxin-NADP reductase | 49.2 | 72.7 | 72.4 | | 0.009468 |
| BMEI2037 | Phosphoenolpyruvate carboxykinase | 113.9 | 155.7 | 158.2 | | 0.021717 |
| BMEII0135 | 5-carboxymethyl-2-hydroxymuconate semialdehyde dehydrogenase | 3.16 | 9.7 | 10.3 | | 0.037007 |
| **TABLE A.1. (continued)** | | | | | | |
|  |  |  |  |  | |  |
| **Locus ID** | **Gene product** | **SAM** | **Spotfire** | **GSpring** | | **ANOVA** |
|  |  |  | **FC** |  | | **(*P*)** |
|  |  |  |  |  | |  |
| BMEII0141 | Aldehyde dehydrogenase | 137.2 | 166.5 | 169.7 | | 0.004314 |
| BMEII0241 | Coniferyl-aldehyde dehydrogenase | 35.8 | 71.0 | 71.6 | | 0.024943 |
| BMEII0246 | Nitroreductase | 78.7 | 78.7 | 78.7 | | 0.000402 |
| BMEII0255 | Iron-sulfur cluster-binding protein | 3.6 | 75.5 | 83.5 | | 0.026113 |
| BMEII0378 | Formate dehydrogenase alpha chain | 214.2 | 214.2 | 214.2 | | 0.005487 |
| BMEII0388 | Piperideine-6-carboxylate dehydrogenase | 5.5 | 106.7 | 101.4 | | 0.007737 |
| BMEII0553 | Alcohol dehydrogenase | -5.3 | -6.6 | -4.8 | | 0.005386 |
| BMEII0588 | Formate dehydrogenase accessory protein | 11.5 | 66.6 | 56.3 | | 0.028185 |
| BMEII0771 | Hydroxyacylglutathione hydrolase | 23.4 | 23.4 | 23.4 | | 0.000994 |
| BMEII0876 | Quinone oxidoreductase | 51.5 | 99.7 | 97.6 | | 0.005751 |
| BMEII0950 | Nitrate reductase alpha chain | 193.9 | 282.4 | 290.3 | | 0.016923 |
| BMEII0951 | Nitrate reductase beta chain | 4.1 | 408.9 | 512.2 | | 0.019189 |
| BMEII0965 | Pseudoazurin | 3.8 | 41.8 | 44.0 | | 0.016079 |
| BMEII0973 | Nitrous-oxide reductase precurser | 24.8 | 43.0 | 42.2 | | 0.03814 |
| BMEII1005 | Malate dehydrogenase | 4.9 | 144.5 | 103.8 | | 0.01867 |
| BMEII1019 | Alpha-methylacyl-CoA racemase | 3.0 | 173.7 | 194.3 | | 0.029976 |
| BMEII1073 | Cytochrome b561 | 31.9 | 31.9 | 31.9 | | 0.009619 |
|  |  |  |  |  | |  |
| **Inorganic ion transport and metabolism** | | | | | | |
| BMEI0317 | Integral membrane protein | 72.2 | 97.4 | 95.2 | | 0.017047 |
| BMEI0450 | Cobalt-Zinc-Cadmium resistance protein CzcD | 5.3 | 8.2 | 7.0 | | 0.009439 |
| BMEI0511 | TRK system potassium uptake protein TrkH | 7.2 | 67.7 | 49.0 | | 0.031708 |
| BMEI0639 | CbiM protein | 124.1 | 275.3 | 278.9 | | 0.003432 |
| BMEI0640 | CbiM protein | -2.6 | -3.0 | -2.7 | | 0.001693 |
| BMEI0660 | Metal chelate transport ATP-binding protein | 2.4 | 2.5 | 2.5 | | 0.007193 |
| BMEI1988 | Phosphate transport system permease protein PstC | -3.9 | -3.9 | -3.7 | | 0.001835 |
| BMEII0338 | ABC transporter substrate binding protein | 4.9 | 98.6 | 96.6 | | 0.001187 |
| BMEII0487 | Nickel-binding periplasmic protein precursor (*nikA*) | 10.2 | 106.7 | 61.5 | | 0.029493 |
| BMEII0488 | Nickel transport system permease protein NikB | 10.0 | 88.4 | 73.1 | | 0.006553 |
| BMEII0536 | Iron(III) dicitrate transport system permease protein FecD | 4.2 | 105.8 | 126.6 | | 0.013862 |
| BMEII0567 | Iron(III)-transport ATP-binding protein SfuC | 7.5 | 383.3 | 497.7 | | 0.022023 |
| BMEII0581 | Superoxide dismutase (Cu-Zn) | 7.4 | 99.2 | 80.5 | | 0.010658 |
| BMEII0704 | Bacterioferritin | 3.2 | 3.5 | 4.0 | | 0.032068 |
| BMEII0765 | Potassium efflux system protein PhaG | 26.9 | 138.0 | 129.4 | | 0.022215 |
| BMEII0767 | Potassium efflux system protein PhaE | 5.3 | 92.0 | 76.3 | | 0.024117 |
| BMEII0883 | High-affinity iron permease | 13.0 | 151.4 | 64.8 | | 0.039901 |
| BMEII1011 | Sulfite reductase (NADPH) flavoprotein alpha-component | 19.9 | 200.1 | 214.6 | | 0.010442 |
| BMEII1120 | Iron(III)-binding periplasmic protein precursor | 20.8 | 131.3 | 137.1 | | 0.016841 |
|  |  |  |  |  | |  |
| **Cofactor transport and metabolism** | | | | | | |
| BMEI0176 | Porphobilinogen deaminase | 4.1 | 33.0 | 41.5 | | 0.006669 |
| **TABLE A.1. (continued)** | | | | | | |
|  |  |  |  |  | |  |
| **Locus ID** | **Gene product** | **SAM** | **Spotfire** | **GSpring** | | **ANOVA** |
|  |  |  | **FC** |  | | **(*P*)** |
|  |  |  |  |  | |  |
| BMEI0177 | Uroporphyrinogen-III synthetase | 2.3 | 2.9 | 2.9 | | 0.02117 |
| BMEI0690 | Cobyric acid synthase | 215.6 | 279.7 | 296.5 | | 0.020047 |
| BMEI0700 | Precorrin-3B C17-methyltransferase | 2.4 | 2.9 | 2.6 | | 0.03227 |
| BMEI0841 | Molybdopterin biosynthesis MoeA protein | -3.0 | -3.2 | -2.7 | | 0.00438 |
| BMEI0842 | Molybdenum cofactor biosynthesis protein C | -5.1 | -6.7 | -4.5 | | 0.007319 |
| BMEI0886 | Phosphopantetheine adenylyltransferase | 73.1 | 73.3 | 73.5 | | 0.010854 |
| BMEI0954 | 2-amino-4-hydroxy-6-hydroxymethyldihydropteridine pyrophosphokinase | 2.3 | 2.5 | 2.4 | | 0.025565 |
| BMEI1187 | Riboflavin synthase subunit beta | 4.1 | 57.9 | 61.1 | | 0.017448 |
| BMEI1517 | Pyridoxamine 5'-phosphate oxidase | 4.4 | 6.0 | 7.8 | | 0.04036 |
| BMEI1592 | 3-methyl-2-oxobutanoate hydroxymethyltransferase | 2.5 | 2.6 | 2.6 | | 0.023893 |
| BMEI1735 | Thiazole synthase | 2.7 | 78.3 | 84.1 | | 0.038439 |
| BMEI1902 | Molybdopterin biosynthesis enzyme | 6.8 | 26.0 | 40.9 | | 0.034044 |
| BMEII0077 | Isochorismate synthase DhbC (*dhbC*) | 17.1 | 70.5 | 58.8 | | 0.02942 |
| BMEII0775 | Biotin synthase | 18.5 | 97.3 | 106.1 | | 0.007341 |
| BMEII0834 | Glutamate-1-semialdehyde 2,1-aminomutase | 2.7 | 4.2 | 4.4 | | 0.019027 |
| BMEII1010 | Thiamine biosynthesis lipoprotein ApbE precursor | 3.2 | 85.8 | 105.2 | | 0.028487 |
| BMEII1044 | Rivoflavin kinase/FMN adenylyltransferase | 5.3 | 139.8 | 147.3 | | 0.00776 |
|  |  |  |  |  | |  |
| **Cell envelope, biogenesis and outer membrane** | | | | | | |
| BMEI0271 | Monofunctional biosynthetic peptidoglycan transglycosylase (*mgtA*) | 7.5 | 75.9 | 56.6 | | 0.028509 |
| BMEI0402 | 31 kDa outer-membrane immunogenic protein precursor | 4.2 | 4.9 | 4.5 | | 0.017121 |
| BMEI0418 | Lipooligosaccharide biosynthesis protein Lic2B | 23.0 | 62.7 | 56.2 | | 0.018425 |
| BMEI0566 | Soluble lytic murein transglycosylase | -3.6 | -4.8 | -3.5 | | 0.001835 |
| BMEI0586 | UDP-3-O-[3-hydroxymyristoyl] N-acetylglucosamine deacetylase | 21.1 | 419.8 | 394.4 | | 0.001476 |
| BMEI0786 | Outer membrane protein | 11.6 | 141.4 | 133.5 | | 0.033327 |
| BMEI0814 | Penicillin-binding protein 6 (D-alanyl-D-alanine carboxypeptidase fraction C) | 99.9 | 151.9 | 141.9 | | 0.032178 |
| BMEI0833 | UDP-N-acetylglucosamine acyltransferase | 70.6 | 145.1 | 156.5 | | 0.00953 |
| BMEI0991 | RarE lipoprotein A | 3.8 | 6.1 | 6.5 | | 0.00441 |
| BMEI1079 | Lipoprotein NlpD | 16.2 | 250.2 | 173.5 | | 0.043447 |
| BMEI1175 | Putative capsule polysaccharide export protein precursor | 6.5 | 193.0 | 176.1 | | 0.020777 |
| BMEI1414 | Perosamine synthetase (*perA*) | 133.4 | 156.9 | 161.9 | | 0.027191 |
| BMEI1493 | Peptidoglycan binding protein (LysM domain) | 3.3 | 55.1 | 61.3 | | 0.036401 |
| BMEI1626 | N-acetylglucosaminyltransferase | 4.2 | 89.4 | 92.6 | | 0.012732 |
| BMEI1707 | Mandelate racemase | 42.5 | 265.7 | 298.4 | | 0.010775 |
| BMEII0083 | Basic membrane protein A precursor | 255.3 | 255.3 | 255.3 | | 0.000461 |
| BMEII0084 | Basic membrane protein A precursor | 156.3 | 156.3 | 156.3 | | 0.006337 |
| BMEII0157 | Hypothetical protein | 69.7 | 99.9 | 99.0 | | 0.011761 |
| BMEII0376 | Heat resistant agglutinin 1 precursor | 100.2 | 100.2 | 100.2 | | 0.023267 |
| BMEII0472 | Membrane fusion protein MtrC | 86.7 | 185.5 | 185.5 | | 0.012122 |
| BMEII0727 | UDP-glucose 6-dehydrogenase | 36.1 | 248.0 | 218.4 | | 0.011001 |
| **TABLE A.1. (continued)** | | | | | | |
|  |  |  |  |  | |  |
| **Locus ID** | **Gene product** | **SAM** | **Spotfire** | **GSpring** | | **ANOVA** |
|  |  |  | **FC** |  | | **(*P*)** |
|  |  |  |  |  | |  |
| BMEII0729 | Cellulose synthase catalytic subunit (UDP-forming) | 89.1 | 106.0 | 109.3 | | 0.002611 |
| BMEII0731 | dTDP-glucose 4-6-dehydratase | 62.1 | 87.4 | 88.3 | | 0.001361 |
| BMEII0836 | dTDP-4-dehydrorhamnose 3,5-epimerase | 53.2 | 53.2 | 53.2 | | 0.026117 |
| BMEII1101 | Bactroprenol glucosyl transferase/Bactoprenol apolipoprotein N-acyltransferase | 4.3 | 190.3 | 160.5 | | 0.026543 |
| BMEII1128 | Succinoglycan biosynthesis protein ExoM | 3.6 | 55.8 | 50.1 | | 0.00868 |
| BMEII1130 | Probable UDP-N-acetyl-D-mannosaminuronic acid transferasa | 76.9 | 83.3 | 83.1 | | 0.002959 |
|  |  |  |  |  | |  |
| **Membrane transport** | | | | | | |
| BMEI0113 | Asparagine transport system permease protein | 73.4 | 73.4 | 73.4 | | 0.003179 |
| BMEI0263 | Leucine-, isoleucine-, valine-, threonine-, and alanine-binding protein precursor | 3.1 | 10.6 | 10.1 | | 0.01319 |
| BMEI0264 | Leucine-, isoleucine-, valine-, threonine-, and alanine-binding protein precursor | 10.3 | 234.4 | 181.7 | | 0.017178 |
| BMEI0642 | Urea transporter | 94.4 | 94.4 | 94.4 | | 0.003138 |
| BMEI0654 | ABC transporter ATP-binding protein | 23.7 | 346.4 | 384.7 | | 0.004751 |
| BMEI0797 | Transporter | 6.9 | 478.8 | 464.2 | | 0.010229 |
| BMEI1022 | Arginine/Ornithine-binding periplasmic protein precursor | 4.2 | 244.4 | 209.7 | | 0.017741 |
| BMEI1041 | ABC transporter ATP-binding protein | -5.0 | -5.5 | -3.9 | | 0.010444 |
| BMEI1416 | O-antigen export system ATP-binding protein RfbB | 207.7 | 207.7 | 207.7 | | 0.015649 |
| BMEI1554 | Transporter, MFS superfamily | 87.2 | 87.2 | 87.2 | | 0.015542 |
| BMEI1580 | Mannitol transporter, large subunit | 3.5 | 69.6 | 72.8 | | 0.017358 |
| BMEI1713 | Maltose/maltodextrin transport ATP-binding protein MalK | 234.4 | 437.5 | 458.8 | | 0.005988 |
| BMEI1912 | Sugar transporter | 44.3 | 89.7 | 87.9 | | 0.015466 |
| BMEII0025 | Attachment mediating protein VirB1 homolog | 9.0 | 115.3 | 109.3 | | 0.010999 |
| BMEII0027 | Channel protein VirB3 homolog | 2.2 | 165.3 | 141.1 | | 0.042572 |
| BMEII0034 | Channel protein VirB10 homolog | 81.6 | 81.6 | 81.6 | | 0.025033 |
| BMEII0038 | D-serine/D-alanine/glycine transporter | 35.03 | 236.2 | 243.8 | | 0.003534 |
| BMEII0066 | High affinity branched-chain amino acid transport ATP-binding protein LivG | 11.7 | 85.3 | 90.2 | | 0.012414 |
| BMEII0087 | Sugar ABC transporter, permease protein | 40.3 | 151.7 | 134.9 | | 0.033397 |
| BMEII0098 | High affinity branched-chain amino acid transport ATP-binding protein LivF | 5.6 | 135.9 | 137.1 | | 0.014354 |
| BMEII0099 | High affinity branched-chain amino acid transport ATP-binding protein LivG | 86.2 | 86.2 | 86.2 | | 0.016303 |
| BMEII0102 | High affinity branched-chain amino acid transport system permease protein LivH | 13.7 | 123.2 | 113.2 | | 0.003106 |
| BMEII0103 | Leu/ile/val-binding protein precursor | 10.2 | 154.4 | 159.9 | | 0.008315 |
| BMEII0114 | Sn-glycerol-3-phosphate transport system permease protein UgpE | 5.9 | 55.6 | 40.7 | | 0.008082 |
| BMEII0144 | Xylose transport system permease protein XylH | 84.1 | 84.1 | 84.1 | | 0.011375 |
| BMEII0196 | Spermidine/putrescine-binding periplasmic protein | 7.7 | 65.8 | 66.1 | | 0.02712 |
| BMEII0200 | Oligopeptide transport ATP-binding protein OppD | 5.8 | 40.8 | 35.4 | | 0.046728 |
| BMEII0223 | Oligopeptide transport ATP-binding protein OppF | 69.4 | 69.4 | 69.4 | | 0.020388 |
| BMEII0284 | Periplasmic dipeptide transport protein precursor | 2.8 | 49.2 | 39.9 | | 0.025605 |
| **TABLE A.1. (continued)** | | | | | | |
|  |  |  |  |  | |  |
| **Locus ID** | **Gene product** | **SAM** | **Spotfire** | **GSpring** | | **ANOVA** |
|  |  |  | **FC** |  | | **(*P*)** |
|  |  |  |  |  | |  |
| BMEII0285 | Dipeptide transport system permease protein DppB | 6.6 | 262.1 | 306.1 | | 0.019232 |
| BMEII0302 | Ribose transport system permease protein RbsC | 3.4 | 44.7 | 25.4 | | 0.028921 |
| BMEII0340 | High affinity branched-chain amino acid transport system permease protein LivM | 4.2 | 4.7 | 4.7 | | 1.00E-04 |
| BMEII0342 | High affinity branched-chain amino acid transport ATP-binding protein LivF | 67.6 | 100.6 | 91.8 | | 0.005617 |
| BMEII0481 | ABC transporter ATP-binding protein | 6.0 | 303.9 | 289.7 | | 0.016367 |
| BMEII0483 | ABC transporter integral membrane protein | 3.0 | 19.3 | 22.0 | | 0.01803 |
| BMEII0517 | Branched-chain amino acid transport protein AzlC | 14.7 | 169.0 | 154.7 | | 0.00594 |
| BMEII0548 | Glycine betaine/L-proline transport ATP-binding protein ProV | 4.1 | 101.4 | 120.6 | | 0.025865 |
| BMEII0583 | Iron(III)-transport ATP-binding protein SfuC | 102.3 | 102.3 | 102.3 | | 0.004557 |
| BMEII0596 | Methylenomycin A resistance protein | 8.1 | 97.8 | 76.0 | | 0.023565 |
| BMEII0618 | Xanthine/uracil permease | 5.7 | 193.7 | 206.2 | | 0.02544 |
| BMEII0621 | Sn-glycerol-3-phosphate transport system permease protein UgpC | 81.4 | 81.4 | 81.4 | | 0.02135 |
| BMEII0622 | Sn-glycerol-3-phosphate transport system permease protein UgpE | 158.6 | 185.6 | 176.9 | | 0.015793 |
| BMEII0623 | Sn-glycerol-3-phosphate transport system permease protein UgpD | -16.2 | -18.0 | -13.9 | | 0.006297 |
| BMEII0624 | Sn-glycerol-3-phosphate transport system permease protein UgpA | 58.3 | 102.1 | 105.0 | | 0.014122 |
| BMEII0628 | High affinity branched-chain amino acid transport ATP-binding protein LivF | 9.0 | 87.8 | 68.7 | | 0.013577 |
| BMEII0662 | Transporter, Msf superfamily | 55.2 | 109.0 | 104.0 | | 0.015113 |
| BMEII0691 | Periplasmic oligopeptide-binding protein precursor | 4.0 | 74.9 | 63.7 | | 0.031048 |
| BMEII0737 | Oligopeptide transport system permease proteinOppC | 3.0 | 88.3 | 70.2 | | 0.012229 |
| BMEII0753 | Sorbitol/mannitol transport inner membrane protein | 3.4 | 42.2 | 45.3 | | 0.039989 |
| BMEII0755 | Sugar-binding protein | 2.8 | 9.0 | 8.6 | | 0.036941 |
| BMEII0845 | Lipopolysaccharide N-acetylglucosaminyltransferase | -2.8 | -3.5 | -2.6 | | 0.014534 |
| BMEII0851 | Exopolysaccharide production protein ExoF precursor | 14.3 | 344.7 | 451.8 | | 0.020084 |
| BMEII0861 | Oligopeptide transport system permease protein AppC | 5.6 | 375.3 | 490.0 | | 0.018617 |
| BMEII0863 | Oligopeptide transport ATP-binding protein AppD | 8.3 | 128.5 | 79.9 | | 0.029032 |
| BMEII0864 | Oligopeptide transport ATP-binding protein AppF | 45.4 | 44.0 | 44.0 | | 0.02127 |
| BMEII0868 | Leucine-specific binding protein precursor | 235.6 | 396.7 | 402.3 | | 0.000378 |
| BMEII0875 | Leucine-specific binding protein precursor | 3.7 | 7.6 | 6.5 | | 0.023208 |
| BMEII0922 | Spermidine/putrescine transport ATP-binding protein PotA | 9.2 | 86.1 | 83.4 | | 0.004857 |
| BMEII1122 | Iron(III)-transport system permease protein SfuB | 71.7 | 71.7 | 71.7 | | 0.003809 |
|  |  |  |  |  | |  |
| **Defense mechanisms** | | | | | | |
| BMEI0403 | Multiple antibiotic resistance protein MarC | 163.0 | 262.8 | 256.8 | | 0.012425 |
| BMEI0656 | Daunorubicin resistance transmembrane protein | 98.95 | 98.95 | 98.95 | | 0.0151 |
| BMEI0893 | Acriflavin resistance protein B | 10.46 | 12.4 | 9.8 | | 0.00035 |
| BMEII0380 | Acriflavin resistance protein A precursor | 267.76 | 267.76 | 267.76 | | 0.009676 |
| BMEII0382 | Acriflavin resistance protein D | 94.95 | 175.5 | 161.3 | | 0.008164 |
| BMEII0451 | Type I restriction-modification system methylation | -3.6 | -3.9 | -3.2 | | 0.007363 |
| **TABLE A.1. (continued)** | | | | | | |
|  |  |  |  |  | |  |
| **Locus ID** | **Gene product** | **SAM** | **Spotfire** | **GSpring** | | **ANOVA** |
|  |  |  | **FC** |  | | **(*P*)** |
|  |  |  |  |  | |  |
| BMEII0533 | Fusaric acid resistance protein FusE | 15.6 | 145.4 | 161.5 | | 0.027832 |
| BMEII0801 | Daunorubicin resistance transmembrane protein | 3.2 | 160.2 | 136.7 | | 0.021329 |
| BMEII0914 | Acriflavin resistance protein A precursor | 5.4 | 130.8 | 161.9 | | 0.030513 |
| BMEII0916 | Acriflavin resistance protein D | 78.66 | 78.66 | 78.66 | | 0.020278 |
|  |  |  |  |  | |  |
| **Signal transduction** | | | | | | |
| BMEI0067 | cAMP-dependent protein kinase regulatory subunit | 2.3 | 2.9 | 2.9 | | 0.00272 |
| BMEI0372 | Sensory transduction regulatory protein | -6.2 | -7.3 | -5.1 | | 0.00684 |
| BMEI0374 | Sensory transduction histidine kinase | -3.8 | -4.4 | -3.6 | | 0.003242 |
| BMEI1328 | Sensory transduction histidine kinase | 3.3 | 131.0 | 122.6 | | 0.030607 |
| BMEI1582 | Transcriptional regulatory protein DegU | 37.7 | 46.7 | 43.8 | | 0.003334 |
| BMEI1606 | Sensory transduction histidine kinase (*vsrB*) | 2.8 | 3.3 | 3.0 | | 0.023505 |
| BMEI2034 | Sensor protein ChvG (*hprK*) | 3.8 | 36.9 | 38.5 | | 0.003834 |
| BMEII0292 | Response regulator protein | 2.9 | 3.6 | 3.5 | | 0.001663 |
| BMEII1027 | Hypothetical protein | 2.4 | 4.1 | 3.9 | | 0.011999 |
|  |  |  |  |  | |  |
| **Post-translational modification and secretion, protein turnover and chaperones** | | | | | | |
| BMEI0643 | Urease accessory protein UreD | 32.4 | 32.4 | 32.4 | | 0.012636 |
| BMEI0644 | Urease accessory protein UreG | 9.3 | 202.1 | 196.6 | | 0.029832 |
| BMEI0645 | Urease accessory protein UreF | 70.1 | 70.1 | 70.1 | | 0.026077 |
| BMEI0646 | Urease accessory protein UreE | 49.7 | 49.7 | 49.7 | | 0.001821 |
| BMEI0783 | Protease DO | 49.6 | 188.8 | 162.3 | | 0.011156 |
| BMEI1080 | Protein-L-isoaspartate O-methyltransferase | -16.3 | -17.0 | -15.7 | | 0.000502 |
| BMEI1331 | Cytochrome C-type biogenesis protein CycL | 4.1 | 158.5 | 164.5 | | 0.005198 |
| BMEI1574 | XdhC protein (assists in molybdopterin insertion into xanthine dehydrogenase) | 4.6 | 110.5 | 107.5 | | 0.000469 |
| BMEI1655 | Urease accessory protein UreD | 15.1 | 371.3 | 391.2 | | 0.037834 |
| BMEI1793 | Putative protease IV | -2.5 | -2.5 | -2.2 | | 0.00705 |
| BMEI1799 | Lipoprotein signal peptidase | 201.8 | 271.9 | 271.6 | | 0.00713 |
|  |  |  |  |  | |  |
| **Cell division** | | | | | | |
| BMEI0008 | Glucose-inhibited division protein B | 8.8 | 291.7 | 360.0 | | 0.017072 |
| BMEI0010 | Chromosome partitioning protein ParB | 8.0 | 116.3 | 106.8 | | 0.000631 |
| BMEI0073 | Cell division protein FtsX | 7.0 | 208.3 | 260.8 | | 4.83E-04 |
| BMEII0470 | Integral membrane protein | 71.7 | 93.4 | 89.1 | | 0.011569 |
| BMEII0925 | Cell division topological specificity factor MinE | 3.5 | 80.7 | 87.7 | | 0.039051 |
|  |  |  |  |  | |  |
| **Cell motility and chemotaxis** | | | | | | |
| BMEI0961 | Kinesin-like protein | 8.8 | 69.1 | 76.0 | | 0.019508 |
| BMEII0150 | Flagellin | 96.6 | 96.6 | 96.6 | | 0.007312 |
| BMEII0151 | Flagellar M-ring protein FliF | 3.1 | 3.7 | 3.3 | | 0.030285 |
| BMEII0156 | Chemotaxis MotD protein | 3.0 | 4.1 | 4.0 | | 0.000409 |
| BMEII0164 | Flagellar basal body rod modification protein | 2.1 | 2.4 | 2.5 | | 0.018005 |
| **TABLE A.1. (continued)** | | | | | | |
|  |  |  |  |  | |  |
| **Locus ID** | **Gene product** | **SAM** | **Spotfire** | **GSpring** | | **ANOVA** |
|  |  |  | **FC** |  | | **(*P*)** |
|  |  |  |  |  | |  |
| BMEII0166 | Flagellar biosynthetic protein FlhA | 5.4 | 104.9 | 97.7 | | 0.034612 |
| BMEII1112 | Flagellar motor switch protein FliN | 10.3 | 139.2 | 130.1 | | 0.003866 |
|  |  |  |  |  | |  |
| **General function prediction only** | | | | | | |
| BMEI0158 | Acetyltransferase | 48.95 | 77.0 | 78.9 | | 0.023737 |
| BMEI0346 | Sodium/bile acid cotransporter homolog, sbf family | 334.6 | 334.6 | 334.6 | | 0.004413 |
| BMEI0350 | Acetyltransferase | 3.8 | 50.6 | 29.4 | | 0.013507 |
| BMEI0594 | Chloramphenicol acetyltransferase | 191.2 | 191.2 | 191.2 | | 0.001931 |
| BMEI0720 | Sugar fermentation stimulation protein | 5.4 | 76.5 | 67.1 | | 0.026151 |
| BMEI0736 | Ferripyochelin binding protein | 4.8 | 200.1 | 195.5 | | 0.022678 |
| BMEI0740 | Inosine-5'-monophosphate dehydrogenase | -7.4 | -8.3 | -6.3 | | 0.008082 |
| BMEI0946 | NAD(FAD)-utilizing dehydrogenase | 4.2 | 15.0 | 15.2 | | 0.004063 |
| BMEI0962 | Membrane lipoprotein lipid attachment SitE containing protein | -20.7 | -267.3 | -16.9 | | 0.004775 |
| BMEI1269 | Chloramphenicol-sensitive protein RarD | 2.4 | 2.9 | 3.0 | | 0.004112 |
| BMEI1370 | ATPase | 3.9 | 57.0 | 61.9 | | 0.025749 |
| BMEI1388 | Oxidoreductase | 4.1 | 88.9 | 75.6 | | 0.018948 |
| BMEI1437 | Putative hydroxilase | 36.7 | 51.8 | 49.4 | | 0.022621 |
| BMEI1443 | 2-Haloalkanoic acid dehalogenase I | 2.3 | 2.7 | 2.8 | | 0.009551 |
| BMEI1487 | Colicin V production protein | 4.25 | 109.6 | 136.5 | | 0.039396 |
| BMEI1534 | Methyltransferase | -5.0 | -5.2 | -4.5 | | 0.005094 |
| BMEI1634 | Phosphoglycolate phosphatase | 30.2 | 83.5 | 93.0 | | 0.02468 |
| BMEI1822 | S-formylglutathione hydrolase | 2.7 | 3.0 | 3.0 | | 0.011448 |
| BMEI2011 | 2-hydroxymuconic semialdehyde hydrolase | 10.3 | 151.7 | 145.9 | | 0.006432 |
| BMEII0327 | Phosphoglycolate phosphatase | 70.5 | 291.1 | 363.5 | | 0.043366 |
| BMEII0829 | Possible S-adenosylmethionine-dependent methyltransferase | 20.0 | 47.3 | 55.5 | | 0.036531 |
| BMEII0838 | Succinoglycan biosynthesis transport protein ExoT | 12.2 | 84.9 | 91.5 | | 0.011502 |
| BMEII1003 | Putative O-antigen transporter | 2.4 | 2.6 | 2.5 | | 0.035181 |
| BMEII1016 | Protease I | 66.5 | 66.5 | 66.5 | | 0.018129 |
| BMEII1019 | Alpha-methylacyl CoA racemase | 3.0 | 173.7 | 194.3 | | 0.029976 |
| BMEII1052 | Transporter | 3.3 | 114.5 | 123.0 | | 0.020979 |
| BMEII1060 | 2,5-diketo-D-glucuronic acid reductase | 7.0 | 237.9 | 189.0 | | 0.01192 |
|  |  |  |  |  | |  |
| **Predicted by homology** | | | | | | |
| BMEI0804 | Hypothetical cytosolic protein | -6.5 | -9.0 | -5.4 | | 0.019652 |
| BMEI1319 | Hypothetical membrane spanning protein | 2.9 | 132.1 | 136.8 | | 0.020961 |
| BMEII0261 | Hypothetical cytosolic protein | 30.8 | 31.7 | 31.7 | | 0.023401 |
| BMEII0522 | Hypothetical protein | 8.1 | 275.0 | 346.4 | | 0.03065 |
|  |  |  |  |  | |  |
| **Unknown** | | | | | | |
| BMEI0002 | Cytochrome functioning/assembly related protein | 3.75 | 218.6 | 242.6 | | 0.026103 |
| BMEI0016 | Hypothetical protein | 3.0 | 7.4 | 9.2 | | 0.035575 |
| **TABLE A.1. (continued)** | | | | | | |
|  |  |  |  |  | |  |
| **Locus ID** | **Gene product** | **SAM** | **Spotfire** | **GSpring** | | **ANOVA** |
|  |  |  | **FC** |  | | **(*P*)** |
|  |  |  |  |  | |  |
| BMEI0018 | Hypothetical cytosolic protein | 2.8 | 3.9 | 3.9 | | 0.001061 |
| BMEI0051 | Hypothetical protein | 29.4 | 29.4 | 29.4 | | 0.010789 |
| BMEI0057 | Hypothetical membrane spanning protein | 2.3 | 2.4 | 2.4 | | 0.014254 |
| BMEI0059 | Hypothetical protein | 13.2 | 66.2 | 60.8 | | 0.018855 |
| BMEI0063 | Hypothetical membrane spanning protein | 4.5 | 108.7 | 102.9 | | 0.030677 |
| BMEI0064 | Hypothetical protein | 7.8 | 209.0 | 237.5 | | 0.02241 |
| BMEI0152 | Hypothetical cytosolic protein | 3.3 | 113.4 | 166.3 | | 0.027048 |
| BMEI0153 | Hypothetical protein | 3.3 | 108.2 | 104.6 | | 0.008451 |
| BMEI0179 | Hypothetical protein | 6.3 | 83.2 | 61.1 | | 0.0013685 |
| BMEI0217 | Hypothetical protein | 6.9 | 218.1 | 235.6 | | 0.009073 |
| BMEI0262 | Hypothetical protein | 2.9 | 106.8 | 126.6 | | 0.026445 |
| BMEI0366 | Hypothetical protein | -3.9 | -4.4 | -3.5 | | 0.00797 |
| BMEI0373 | Hypothetical protein | -3.5 | -3.8 | -3.3 | | 0.005577 |
| BMEI0422 | Hypothetical protein | 3.4 | 14.7 | 10.9 | | 0.00701 |
| BMEI0425 | Hypothetical protein | 4.0 | 41.8 | 29.3 | | 0.00842 |
| BMEI0431 | Hypothetical protein | 18.9 | 43.3 | 46.6 | | 0.034105 |
| BMEI0442 | Hypothetical protein | 112.1 | 222.9 | 214.6 | | 0.005347 |
| BMEI0448 | Hypothetical protein | 68.1 | 83.6 | 86.3 | | 0.02205 |
| BMEI0458 | Hypothetical membrane spanning protein | 26.3 | 195.3 | 182.2 | | 0.007626 |
| BMEI0498 | Cold shock protein CspA | 3.2 | 3.6 | 4.0 | | 0.031073 |
| BMEI0542 | Hypothetical protein | 3.7 | 68.1 | 60.6 | | 0.015161 |
| BMEI0550 | Hypothetical protein | 231.3 | 231.3 | 231.3 | | 2.70E-05 |
| BMEI0590 | Hypothetical protein | 72.3 | 250.8 | 259.7 | | 0.005759 |
| BMEI0600 | Hypothetical membrane spanning protein | 3.6 | 83.5 | 76.9 | | 0.012201 |
| BMEI0601 | Hypothetical protein | 5.7 | 100.2 | 80.3 | | 0.039441 |
| BMEI0607 | Hypothetical cytosolic protein | 4.5 | 73.8 | 55.8 | | 0.034613 |
| BMEI0620 | Hypothetical protein | -6.5 | -7.6 | -4.7 | | 0.01773 |
| BMEI0638 | Hypothetical protein | 32.6 | 237.6 | 217.3 | | 0.008528 |
| BMEI0678 | Low pH-induced protein A | -4.1 | -6.0 | -3.3 | | 0.012169 |
| BMEI0692 | Hypothetical protein | 8.9 | 390.0 | 383.9 | | 0.000643 |
| BMEI0798 | Hypothetical protein | -15.4 | -18.0 | -15.4 | | 0.001644 |
| BMEI0805 | Hypothetical protein | -36.4 | -56.4 | -26.5 | | 0.003588 |
| BMEI0813 | Hypothetical protein | -9.2 | -11.1 | -8.5 | | 0.001088 |
| BMEI0903 | Hypothetical protein | 4.5 | 93.7 | 84.9 | | 0.019457 |
| BMEI1026 | Outer membrane protein E | 2.8 | 3.7 | 3.9 | | 0.023952 |
| BMEI1028 | Hypothetical protein | 8.1 | 186.3 | 227.3 | | 0.024455 |
| BMEI1072 | Hypothetical protein | -25.8 | -29.3 | -17.8 | | 0.007248 |
| BMEI1086 | Hypothetical cytosolic protein | 2.9 | 4.2 | 4.2 | | 0.021914 |
| BMEI1165 | Hypothetical membrane spanning protein | 28.6 | 30.2 | 30.2 | | 0.004184 |
| BMEI1173 | Hypothetical membrane spanning protein | -2.8 | -3.0 | -2.8 | | 1.92E-04 |
| BMEI1214 | Hypothetical protein | -5.4 | -5.5 | -4.9 | | 0.009011 |
| BMEI1219 | Hypothetical protein | 5.0 | 467.0 | 572.7 | | 0.024323 |
| **TABLE A.1. (continued)** | | | | | | |
|  |  |  |  |  | |  |
| **Locus ID** | **Gene product** | **SAM** | **Spotfire** | **GSpring** | | **ANOVA** |
|  |  |  | **FC** |  | | **(*P*)** |
|  |  |  |  |  | |  |
| BMEI1242 | Hypothetical membrane spanning protein | -7.6 | -10.9 | -5.7 | | 0.009799 |
| BMEI1275 | Hypothetical protein | 3.0 | 37.2 | 35.4 | | 1.10E-02 |
| BMEI1298 | Hypothetical cytosolic protein | 11.8 | 199.6 | 187.5 | | 0.008254 |
| BMEI1317 | Hypothetical protein | 23.0 | 116.3 | 130.9 | | 0.013377 |
| BMEI1358 | Hypothetical cytosolic protein | 121.1 | 121.1 | 121.1 | | 0.005318 |
| BMEI1361 | Hypothetical cytosolic protein | 12.9 | 177.6 | 168.0 | | 0.020648 |
| BMEI1371 | Hypothetical protein | 4.8 | 148.3 | 119.3 | | 0.041039 |
| BMEI1417 | Perosamine synthetase WbkB | 225.2 | 338.0 | 140.2 | | 0.017503 |
| BMEI1428 | Ribonuclease III | 3.1 | 156.8 | 172.5 | | 0.031964 |
| BMEI1431 | BioY protein | 19.2 | 19.2 | 19.2 | | 0.008123 |
| BMEI1461 | Zinc-finger protein | 79.4 | 79.4 | 79.4 | | 0.015544 |
| BMEI1474 | Hypothetical protein | 2.8 | 2.9 | 2.8 | | 0.022541 |
| BMEI1507 | Hypothetical protein | -4.6 | -5.3 | -4.7 | | 0.000656 |
| BMEI1508 | Putative lipoprotein | 2.5 | 99.7 | 94.9 | | 0.041956 |
| BMEI1509 | Hypothetical protein | 3.1 | 95.9 | 91.2 | | 0.03679 |
| BMEI1516 | Hypothetical protein | 20.4 | 20.4 | 20.4 | | 1.18E-02 |
| BMEI1538 | Hypothetical protein | 146.3 | 157.0 | 157.6 | | 0.001598 |
| BMEI1572 | Hypothetical membrane spanning protein | 49.1 | 87.2 | 70.7 | | 0.014322 |
| BMEI1665 | Hypothetical protein | 47.1 | 62.9 | 62.9 | | 0.018253 |
| BMEI1673 | Zinc-binding protein | 4.7 | 158.4 | 176.8 | | 0.00935 |
| BMEI1681 | Hypothetical protein | 2.7 | 86.9 | 108.5 | | 0.017799 |
| BMEI1699 | Hypothetical protein | 5.9 | 119.7 | 105.4 | | 0.014791 |
| BMEI1711 | Hypothetical protein | 187.0 | 187.0 | 187.0 | | 0.008656 |
| BMEI1761 | Hypothetical protein | 11.4 | 164.2 | 155.7 | | 0.001759 |
| BMEI1767 | Hypothetical protein | 3.3 | 22.3 | 21.0 | | 0.04257 |
| BMEI1785 | Hypothetical protein | -4.6 | -7.9 | -4.3 | | 0.004907 |
| BMEI1795 | Hypothetical protein | 4.8 | 199.7 | 268.0 | | 0.02577 |
| BMEI1857 | Hypothetical cytosolic protein | 4.4 | 137.1 | 153.5 | | 0.0001582 |
| BMEI1866 | Hypothetical protein | 5.1 | 98.2 | 97.9 | | 0.035802 |
| BMEI1893 | Protein YbiS precursor | 148.6 | 148.6 | 148.6 | | 0.010974 |
| BMEI1993 | Hypothetical exported protein | 23.2 | 190.5 | 174.4 | | 0.023201 |
| BMEI2044 | Hypothetical membrane spanning protein | -2.3 | -2.3 | -2.1 | | 0.006975 |
| BMEI2049 | Hypothetical protein | 12.5 | 151.5 | 135.2 | | 0.036594 |
| BMEII0043 | Hypothetical protein | 38.6 | 102.5 | 81.5 | | 0.030811 |
| BMEII0082 | Hypothetical protein | 201.8 | 201.8 | 201.8 | | 0.001519 |
| BMEII0090 | Hypothetical protein | 32.8 | 32.8 | 32.8 | | 0.018867 |
| BMEII0094 | Hypothetical protein | 7.2 | 310.1 | 306.7 | | 0.006073 |
| BMEII0231 | SlyX protein | 6.9 | 510.9 | 649.5 | | 0.016558 |
| BMEII0237 | Hypothetical protein | 82.3 | 86.6 | 82.2 | | 0.026051 |
| BMEII0296 | Hypothetical protein | 26.6 | 158.7 | 145.5 | | 0.000888 |
| BMEII0399 | Hypothetical protein | 69.2 | 90.9 | 91.9 | | 0.005163 |
| BMEII0529 | Surface protein | 2.5 | 3.0 | 3.5 | | 0.039543 |
| **TABLE A.1. (continued)** | | | | | | |
|  |  |  |  |  | |  |
| **Locus ID** | **Gene product** | **SAM** | **Spotfire** | **GSpring** | | **ANOVA** |
|  |  |  | **FC** |  | | **(*P*)** |
|  |  |  |  |  | |  |
| BMEII0615 | Hypothetical protein | -60.5 | -70.5 | -43.9 | | 0.010396 |
| BMEII0658 | Hypothetical protein | 15.3 | 193.4 | 179.1 | | 0.004162 |
| BMEII0668 | Putative integral membrane protein | 4.3 | 103.3 | 129.3 | | 0.022889 |
| BMEII0682 | Oxacillin resistance-associated protein FmtC | 4.7 | 49.2 | 43.4 | | 0.027366 |
| BMEII0733 | Hypothetical protein | 2.3 | 3.2 | 3.1 | | 0.039316 |
| BMEII0805 | Hypothetical protein | 4.0 | 140.1 | 116.1 | | 0.025098 |
| BMEII0905 | Hypothetical protein | 224.3 | 224.3 | 224.3 | | 0.00956 |
| BMEII0935 | Nickel resistance protein | 2.7 | 8.2 | 10.1 | | 0.021854 |
| BMEII0993 | Hypothetical protein | 30.3 | 30.0 | 30.0 | | 0.033095 |
| BMEII0994 | Hypothetical protein | 3.3 | 12.1 | 9.0 | | 0.011808 |
| BMEII0995 | Hypothetical protein | 3.1 | 360.2 | 290.1 | | 0.023106 |
| BMEII1013 | Hypothetical cytosolic protein | 5.8 | 243.9 | 262.2 | | 0.008494 |
| BMEII1091 | Hypothetical pyridoxal phosphate biosynthesis protein | 108.1 | 206.2 | 175.9 | | 0.01675 |
|  |  |  |  |  | |  |

FC = Fold-change

Negative sign (-) before the number indicates down-regulation of the gene

GSpring = GeneSpring software

SAM = Significance Analysis of Microarrays software

Spotfire = Spotfire DecisionSite 8.2 software

ANOVA = Analysis of variance
