# Supplementary material for: Brucella melitensis global gene expression study provides novel information on growth phase-specific gene regulation with potential insights for understanding Brucella:host initial interactions
Source: BMC Microbiol. 2009 May 6;9:81. doi: 10.1186/1471-2180-9-81 (PMC2684542; doi:10.1186/1471-2180-9-81)
Supplement: Additional file 3 — Hierarchical cluster of genes from B. melitensis grown to stationary and late-log phases. Hierarchical clustering was performed on normalized Cy3 (transcript) signal intensity values from 8 arrays using Spotfire DecisionSite 8.2 software. Columns represent samples, and rows represent individual probes/genes. Higher signal values are shown in red, and lower signal values are shown in green. Note that all four stationary phase samples clustered together and apart from all four log phase cultures (tick line indicates individual growth phase replicate). Numbers in the top left of the figure indicate the number of cluster levels. The number below (-0.913) represents the calculated similarity measure between the two subnodes in each node. [file 1471-2180-9-81-S3.doc]

# Additional file 3
