# Supplementary material for: Brucella melitensis global gene expression study provides novel information on growth phase-specific gene regulation with potential insights for understanding Brucella:host initial interactions
Source: BMC Microbiol. 2009 May 6;9:81. doi: 10.1186/1471-2180-9-81 (PMC2684542; doi:10.1186/1471-2180-9-81)
Supplement: Additional file 4 — RT-PCR primers. The table describes the primers used for testing B. melitensis gene expression by Real time – PCR. [file 1471-2180-9-81-S4.doc]

Additional file 4. RT-PCR primers. Primers for Real time – PCR tested genes in *B. melitensis*

| **Functional categories** | **Locus ID** | **Gene product** | **Forward primers (5'-3')** | **Reverse primers (5'-3')** |
| --- | --- | --- | --- | --- |
|  |  |  |  |  |
| Cell division | BMEI0073 | Cell division protein FtsX | CATCGAGGTGCTGCATTTCAT | ATAGGATGCCCACCAGGAGAA |
| Carbohydrate metabolism | BMEI0344 | Phosphoglucosamine mutase/phosphoacetylglucosamine mutase/phosphomannomutase | CTTATCTCCGCGCTCCAGAT | CTGATTTCACGCGTTTGTTTTC |
| Cell envelope | BMEI0402 | 31 KDA outer-membrane immunogenic protein precursor | GGCTTCACCCCGACTGAAC | GTTGGTGACGGCGTATTCTACA |
| Energy production | BMEI0475 | Cytochrome C1 | GCTGCAGCGGCTAATAATGG | CGGTCAAAAGCGAATGGATATAA |
| Nucleotide metabolism | BMEI0608 | Thymidylate synthase | TGCCCTGTTGACGATGATGA | ATGCATCACCGGCAGCTT |
| Membrane transport | BMEI0642 | Urea transporter | GTTTCTGGTCCTTGCAGCCTAT | ACGGCATTGTTGAGGAGGAA |
| Post-translational modification | BMEI0645 | Urease accessory protein UreF | AGCCTCGGGCTTGCTTTT | TCGCCTCCAGCAGAATTTTT |
| AA metabolism | BMEI0730 | Lactoylglutathione lyase | CAGCCCCCTCCGATACAGAT | AGCTTCTCGCAGGTGGCATA |
| Cofactor transport & metabolism | BMEI0842 | Molybdenum cofactor biosynthesis protein C | CGCGCACTAGCCCGATT | CGCCGCTTTTTTCGATCA |
| Transcription | BMEI1384 | Transcriptional regulator, AraC family | CGCAGTTCACCAAGGCATT | GCGTGTTCAGAGGCGATCTT |
| Translation | BMEI1798 | 23S ribosomal RNA methyltransferase | CATGGGCTCGGTCTTTTCC | TGTTCATTGCCCATTATCAGGAT |
| Signal transduction | BMEI2034 | Sensor protein ChvG | GCCTGTTCCGCATTCCCTAT | GCGCATTGGAATCACCATTT |
| Lipid metabolism | BMEII0047 | Lysophospholipase L2 | GGCTATGTGCGCAGCTTCA | GGGAGGAGAGCCGTTCCA |
| Secondary metabolism | BMEII0079 | Isochorismatase | CGCCTTGCGCTGAAATATGT | GCGAATCGAGGCCGTAGAG |
| Cell motility | BMEII0150 | Flagellin | GCGGTTGACAAGATCACTGTCA | CGAGAGAAGCAAGAGCGGTTT |
| Defense mechanism | BMEII0382 | Acriflavin resistance protein D | TTGCAGGATCAGAATGCGATT | CATCCGACAGGCGGAAGA |
| DNA replication & repair | BMEII0663 | Phosphohydrolase (MutT/NUDIX family protein) | CGGAGGTGGAGACCCAGAA | TCAAGCTGCGTATCCATCAAAA |
| Inorganic ion transport | BMEII1120 | Iron(III)-binding periplasmic protein precursor | GCAAGAAGGGCCTCGAATTC | TTCGGGATGATGGTTTCCA |
| Control | AF220147 | rRNA 16S | CCTTACGGGCTGGGCTACA | TGATCCGCGATTACTAGCGATT |
|  |  |  |  |  |
